# Targeting galectin-3 to counteract spike-phase uncoupling of fast-spiking interneurons to gamma oscillations in Alzheimer’s disease

**DOI:** 10.1186/s40035-023-00338-0

**Published:** 2023-02-06

**Authors:** Luis Enrique Arroyo-García, Sara Bachiller, Rocío Ruiz, Antonio Boza-Serrano, Antonio Rodríguez-Moreno, Tomas Deierborg, Yuniesky Andrade-Talavera, André Fisahn

**Affiliations:** 1 Neuronal Oscillations Laboratory, Division of Neurogeriatrics, Center for Alzheimer Research, Department of Neurobiology, Care Sciences and Society, Karolinska Institutet, 17164 Solna, Sweden; 2 Experimental Neuroinflammation Laboratory, Department of Experimental Medical Science, Lund University, BMC B11, 221 84 Lund, Sweden; 3 Clinical Unit of Infectious Diseases, Microbiology and Parasitology, Institute of Biomedicine of Seville (IBiS), Virgen del Rocío University Hospital, CSIC, University of Seville, Seville, Spain; 4 Department of Biochemistry and Molecular Biology, University of Seville, Calle Profesor García González Nº2, 41012 Seville, Spain; 5 Laboratory of Cellular Neuroscience and Plasticity, Department of Physiology, Anatomy and Cellular Biology, Universidad Pablo de Olavide, Carretera de Utrera Km-1, 41013 Seville, Spain; 6 Department of Biosciences and Nutrition, Neo, Karolinska Institutet, 141 83 Huddinge, Sweden

**Keywords:** Galectin-3, Gamma oscillations, Neuronal network dynamics, Fast-spiking interneurons, Alzheimer’s disease models, Neuroinflammation, TD139, Hippocampus

## Abstract

**Background:**

Alzheimer’s disease (AD) is a progressive multifaceted neurodegenerative disorder for which no disease-modifying treatment exists. Neuroinflammation is central to the pathology progression, with evidence suggesting that microglia-released galectin-3 (gal3) plays a pivotal role by amplifying neuroinflammation in AD. However, the possible involvement of gal3 in the disruption of neuronal network oscillations typical of AD remains unknown.

**Methods:**

Here, we investigated the functional implications of gal3 signaling on experimentally induced gamma oscillations ex vivo (20–80 Hz) by performing electrophysiological recordings in the hippocampal CA3 area of wild-type (WT) mice and of the 5×FAD mouse model of AD. In addition, the recorded slices from WT mice under acute gal3 application were analyzed with RT-qPCR to detect expression of some neuroinflammation-related genes, and amyloid-β (Aβ) plaque load was quantified by immunostaining in the CA3 area of 6-month-old 5×FAD mice with or without Gal3 knockout (KO).

**Results:**

Gal3 application decreased gamma oscillation power and rhythmicity in an activity-dependent manner, which was accompanied by impairment of cellular dynamics in fast-spiking interneurons (FSNs) and pyramidal cells. We found that the gal3-induced disruption was mediated by the gal3 carbohydrate-recognition domain and prevented by the gal3 inhibitor TD139, which also prevented Aβ42-induced degradation of gamma oscillations. Furthermore, the 5×FAD mice lacking gal3 (5×FAD-Gal3KO) exhibited WT-like gamma network dynamics and decreased Aβ plaque load.

**Conclusions:**

We report for the first time that gal3 impairs neuronal network dynamics by spike-phase uncoupling of FSNs, inducing a network performance collapse. Moreover, our findings suggest gal3 inhibition as a potential therapeutic strategy to counteract the neuronal network instability typical of AD and other neurological disorders encompassing neuroinflammation and cognitive decline.

**Supplementary Information:**

The online version contains supplementary material available at 10.1186/s40035-023-00338-0.

## Background

Neuronal rhythmic electrical activity in the gamma-frequency band (⁓30–80 Hz; gamma oscillations) underlies cognitive processes such as memory storage and recall and plays a role in sensory perception [1–5]. This cognition-relevant brain rhythm emerges from the finely balanced and cooperative activity of particular neuronal populations within neuronal networks. As such, gamma oscillations depend on recurrent activity between excitatory pyramidal cells (PCs) [6] and the pacing of their activity by fast-spiking perisomatic GABAergic interneurons that express parvalbumin [7–9]. Inhibitory (GABAergic) activity has been related to cognitive processes, including working memory, formation and consolidation of fear conditioning, and thought suppression, indicating a crucial role of GABAergic interneurons [10]. The synchronized interplay between PCs and fast-spiking interneurons (FSNs) generates and depends on the finely-patterned fluctuations of excitatory and inhibitory currents that give rise to oscillations of the local field potential [11, 12]. The gamma rhythm is prominent in the hippocampus, where its disruption has been reported to be linked to Alzheimer’s disease (AD) [13–16]. Particularly, reduced levels of GABA in different brain areas of AD patients, including the hipocampus, the cingulate cortex, the amygdala, and the frontal and temporal cortices have been found (see ref. [10] for review).

AD is a neurodegenerative disease characterized by diverse cellular and molecular pathological events that progressively lead to cognitive impairment. Such pathological events mainly, but not exclusively, comprise abnormal extracellular accumulation of beta-amyloid peptide (Aβ42) [17–19], intraneuronal depositions of abnormally phosphorylated tau-tangles [20, 21], defective synaptic transmission, and synaptic and neuronal loss, leading to marked instability of neuronal network and deficient information processing [22]. In this regard, studies performed in AD models suggest a dynamic nature of synaptic and intrinsic dysfunctions destabilizing spontaneous spiking activity of diverse neuronal classes in the hippocampus and cortex, leading to aberrant activity of cortico-hippocampal circuits [22].

There is growing evidence that in AD, synaptic failure and impaired network performance far precede Aβ plaque deposition and clinical expression of cognitive decline [23–25]. Interestingly, diverse studies suggest that neuroinflammation, and specifically myeloid cells, play a major role in the initiation and progression of AD [26–30] with a negative impact on brain circuit functioning [31, 32]. Recently, we found galectin-3 (gal3) to be a central regulator of microglial immune response in Parkinson’s disease [33, 34] and in prolonging sustained microglial activation in AD [27]. As such, microglia, the resident phagocytic immune myeloid cells of the central nervous system, are a recognized part of the cellular phase of AD [35].

Microglial activation has been reported to strongly correlate with amyloid deposition in patients with mild cognitive impairment [36], and activated microglia have been found surrounding amyloid plaques [27, 37, 38] and neurofibrillary tangles [36, 39, 40], probably in an attempt to phagocytose them. However, their activation in AD becomes persistent and, eventually, ineffective [41, 42]. Microglia-driven inflammatory responses include gal3 release, which has emerged as one of the foremost molecules in brain innate immunity associated with neurodegeneration [27, 43]. Gal3 belongs to a protein family with at least 15 members that have substantial sequence similarity in their carbohydrate-recognition domain (CRD). It binds to β-galactosides with variable affinities and specificities [44, 45] and has been found associated with microglial cells in close vicinity to Aβ42-containing plaques in AD patients as well as in animal and cellular models [27].

Gal3 has been found to act as an endogenous ligand for paracrine/autocrine toll-like receptor 4 (TLR4) as well as the triggering receptor expressed on myeloid cells 2 (TREM2), binding through its CRD [27, 33]. Moreover, gal3 has been found significantly upregulated in AD patients [27], surrounding the atherosclerotic plaques in relation to atherogenesis [46], and associated with other human diseases such as major kidney adverse events [47], stroke [48] and atopic dermatitis and psoriasis [49]. Notably, it has been observed that gal3 inhibition decreases the inflammatory response of microglial cells to treatment with fibrillar Aβ. In the same study, a significant Aβ plaque reduction and improvement of cognitive performance were observed in 5×FAD mice lacking gal3 [27]. Also, gal3 is capable of binding TREM2, a central microglial receptor important in AD and essential for a functional microglial response [27]. However, possible functional involvement of gal3 in the disruption of cognition-relevant neuronal network oscillations remains unknown.

Here, we set out to perform ex vivo recordings of gamma oscillations in the cognition-relevant hippocampal CA3 area [50] of wild-type (WT) and 5×FAD mouse brain slices. Gamma oscillations were induced by applying 100 nM kainic acid (KA) either in an interface- or a submerged-type recording chamber. Concomitantly to local field potential (LFP) recordings, we performed whole-cell patch-clamp recordings of FSNs and PCs with emphasis on FSN activity.

## Materials and methods

### Animals

Experiments were performed in accordance with the ethical permit granted by Norra Stockholm’s Djurförsöksetiska Nämnd (dnr N45/13) to AF and Malmö-Lund Ethical Committee on Animal Testing in Sweden (Dnr 5.8.18–01107/2018) to TD. For electrophysiological studies performed under acute application of compounds to WT brain slices, C57/BL6 male mice supplied by Charles River (Erkrath, Germany), were used on postnatal days 18–28. 5×FAD transgenic mice were acquired originally from Jackson Laboratories (Bar Harbor, ME), and crossbred at Lund University Animal Facility and were provided by TD and SB for electrophysiological studies. Gal3KO mice were provided by Dr. K. Sävman from Gothenburg University and crossbred at Lund’s animal facility in homozygosis. 5×FAD-Gal3KO mice were generated by crossing heterozygous 5×FAD male mice with homozygous Gal3KO females to generate 5×FAD^+/−^-Gal3^+/−^ mice. Subsequent crossings between animals expressing this genotype allowed for the generation of 5×FAD-Gal3KO mice. Thus, as in Boza-Serrano et al. [27], 5×FAD and WT mice were littermates, and 5×FAD-Gal3KO and Gal3KO were also littermates; all were used at age of 6 months. Ear biopsies were collected at the time of weaning on postnatal day 30. Mice were group-housed (4–5 animals/cage) in the Innovive disposable individually ventilated cage system (San Diego, CA) with water, food and nesting material provided *ad libitum* under a 12-h light/dark cycle.

### Genotyping

Mice were genotyped for WT, 5×FAD or Gal3 by PCR as described previously [27]. Briefly, DNA from ear punches was extracted using the extraction kit (Extract-N-Amp™, Sigma-Aldrich, Schnelldorf, Germany) and amplified by PCR using the 2× PCR Bio HS Taq Mix Red enzyme (PCR Biosystems, Techtum, Nacka, Sweden), with the following primers (5′–3′): For 5×FAD mice: *App* (Forward AGGACTGACCACTCGACCAG; Reverse CGGGGGTCTAGTTCTGCAT); *Psn1* (Forward AATAGAGAACGGCAGGAGCA; Reverse GCCATGAGGGCACTAATCAT; WT: *App* (Forward CTAGGCCACAGAATTGAAAGATCT; Reverse GTAGGTGGAAATTCTAGCATCATCC). For Gal3KO mice: *Gal3* (Forward: CACGAACGTCTTTTGCTCTCTGG); *Gal3*^*−/−*^ Reverse: GCTTTTCTGGATTCATCGACTGTGG; *Gal3*^*+/+*^ Reverse: TGAAATACTTACCGAAAAGCTGTCTGC.

### RNA extraction and RT-qPCR analysis

Total RNA from frozen brain sections was extracted using TRI-reagent protocol (Sigma-Aldrich) following the manufacturer’s instructions. RNA concentrations were measured using NanoDrop (2000 C, Fisher Scientific, Göteborg, Sweden), and 1 µg of total RNA was converted to cDNA using iScript™ cDNA synthesis kit (BioRad, Solna, Sweden). Real-time RT-qPCR was performed with SensiFAST™ SYBR No-ROX kit (Bioline) and 0.4 µM of the following primer sequences (5’-3’): *Trem2* (Forward: GTTTCTTGCAGCCAGCATCC; Reverse: GGGTCCAGTGAGGATCTGAAG); *Tlr4* (Forward: GGCATCATCTTCATTGTCC; Reverse: TCGAGGCTTTTCCATCCAA); *Clec7a* (Forward: CTGGTATGGAAGTAAGAGACACTGC; Reverse: CGGTGAGACGATGTTTGGC); *Cx3cr1* (Forward: CAGCATCGACCGGTACCTT; Reverse: GCTGCACTGTCCGGTTGTT); and *Gfap* (Forward: TCCTGGAACAGCAAAACAAG; Reverse: CAGCCTCAGGTTGGTTTCAT). Amplification was done in the CFX96™ Real-Time System-C1000™ Thermal Cycler (BioRad) at 95 °C for 2 min, followed by 40 cycles at 95 °C for 5 s and 60 °C for 15 s. Relative gene expression was represented as ∆Ct, normalized to *Gapdh* (Forward: ACCCAGAAGACTGTGGATGG; Reverse: ACACATTGGGGGTAGGAACA).

### Hippocampal slice preparation

Hippocampal slices were obtained as previously described [51–53]. For brain dissection, mice were deeply anaesthetized with isofluorane (2%) before being sacrificed by decapitation. In brief, after mouse sacrifice, the brain was quickly dissected out and placed in ice-cold artificial cerebrospinal fluid (ACSF) slightly modified for dissection, containing (in mM): 80 NaCl, 24 NaHCO_3_, 25 glucose, 1.25 NaH_2_PO_4_, 1 ascorbic acid, 3 Na﻿+-pyruvate, 2.5 KCl, 4 MgCl_2_, 0.5 CaCl_2_, and 75 sucrose, and continuously bubbled with carbogen (95% O_2_ and 5% CO_2_). Horizontal hippocampal sections (350-µm thick) were obtained from both hemispheres with a Leica VT1200S vibratome (Leica Microsystems, Stockholm, Sweden). After slicing, hippocampal slices were collected and allowed to recover in an interface recovery/holding chamber containing standard recording ACSF containing (in mM): 124 NaCl, 30 NaHCO_3_, 10 glucose, 1.25 NaH_2_PO_4_, 3.5 KCl, 1.5 MgCl_2_, and 1.5 CaCl_2_. The holding chamber was continuously supplied with humidified carbogen gas (5% CO_2_, 95% O_2_), held at 37 °C during slicing and then allowed to cool down to room temperature for at least 1 h prior to start of any experiment.

### Electrophysiological recordings

LFP recordings were performed in either interface- or submerged-type recording chambers in the hippocampal CA3 area using borosilicate glass microelectrodes filled with standard ACSF and placed in the *stratum pyramidale*. For LFP recordings in an interface configuration, slices were continuously supplied with aerated ACSF at a rate of 4.5 ml/min at 34 °C and the chamber was continuously supplied with humidified carbogen gas. Single-cell recordings were carried out either concomitantly with LFP or on their own in a submerged recording chamber continuously supplied with aerated ACSF at a perfusion rate of 3 ml/min at 34 °C. Depending on the configuration of patch-recordings (voltage- or current-clamp), different intracellular solutions were used. Action potentials (AP) and excitatory postsynaptic currents (EPSCs) from PCs and FSNs were recorded in whole-cell mode with an internal recording solution for current-clamp configuration containing (in mM): 122.5 K^+^-gluconate, 8 KCl, 2 Mg^2+^-ATP, 0.3 Na^+^-GTP, 10 HEPES, 0.2 EGTA, and 2 MgCl_2_. An internal recording solution for voltage-clamp containing (in mM) 140 CsMetSO_4_, 2 Mg^2+^ ATP, 0.3 Na^+^-GTP, 10 HEPES, and 0.6 EGTA was used for recordings of PC inhibitory postsynaptic currents (IPSCs). For each internal solution pH was set to 7.2–7.3 and osmolarity to 270–280 mosmol/l. EPSCs of PCs and FSNs, and IPSCs of PCs, were recorded in voltage-clamp configuration holding the membrane potential at − 70 mV and 0 mV, respectively. FSN membrane potential (Em) and firing threshold were recorded in current-clamp configuration at resting membrane potential in gap-free for Em, or by applying current steps of 10 pA starting at − 70 mV holding membrane potential for firing threshold.

PCs and FSNs were visually identified under an upright microscope using IR-DIC microscopy (Axioskop, Carl Zeis AG, Göttingen, Germany) based on their morphology and location in the hippocampal *str. pyramidale* and *str. radiatum*, respectively. PCs and the interneuron subtype (FSN) were further confirmed as previously described by applying different stimulation protocols to verify the cellular population type by their unique electrophysiological characteristics (Additional file 1: Fig. S1) [25, 52, 54, 55]. For recordings of PC or FSN in whole-cell configuration access resistance was monitored throughout the experiment. Cells were excluded from the study if more than 20% of change of the access resistance was observed.

Gamma oscillations were induced by applying 100 nM KA (Tocris, Bristol, UK) [51, 52] or 10 µM acetylcholine (ACh) and 2 µM of the acetylcholine esterase inhibitor physostigmine (Phys) [56], and allowed to stabilize prior to recording of any cellular/LFP parameter. LFPs were recorded either in an interface-type recording chamber or concomitantly with whole-cell patch-clamp recordings. LFP recordings in the interface-type recording chamber were acquired with a 4-channel M102 amplifier (University of Cologne, Germany). Data were sampled at 10 kHz, conditioned using a HumBug 50-Hz noise eliminator (Quest Scientific, North Vancouver, BC, Canada), low-pass filtered at 1 kHz, digitized using a Digidata 1440 A (Molecular Devices, CA) and stored using pCLAMP 9.6 software (Molecular Devices). LFP and patch-clamp recordings in submerged-type recording chamber were acquired with a patch-clamp amplifier (Multiclamp 700B) using pCLAMP 10.4 software (Molecular Devices). LFPs recorded in the submerged-type recording chamber were also conditioned using a HumBug 50 Hz noise eliminator (Quest Scientific). All signals recorded in the submerged configuration were low-pass filtered at 1 kHz, acquired at 5 kHz, digitized and stored using Digidata 1322 A and pCLAMP 10.4 softwares (Molecular Devices).

### Immunofluorescence

5×FAD and 5×FAD-Gal3KO mice of 6 months old were anesthetized (isofluorane) and transcardially perfused with 4% paraformaldehyde and PBS, pH 7.4. Brains were removed, cryoprotected in sucrose and frozen in isopentane at − 15 °C. Serial coronal sections (30-µm thick) were cut with a cryostat, and further processed. The staining protocol had been reported previously [27]. Briefly, brain sections were permeabilized with 1% (*v*/*v*) Triton X-100 in PBS for 1 h, and then incubated in 5% (*w*/*v*) BSA in PBS containing 1% Triton X-100 for 1 h, to prevent unspecific staining. The tissue was then incubated overnight at 4 ºC with primary antibody against Aβ (1:1000, Sigma-Aldrich, A3981), gal3 (1:1000, R&D Systems, Minneapolis, MN, AF1197) or Iba 1 (1:500, WAKO, Chuo-Ku, Osaka, Japan, #019-19741), followed by rinses for 1 h in PBS containing 0.1% Triton X-100. After incubation for 1 h with the corresponding secondary antibodies (1:500; Alexa antibodies, Invitrogen, Waltham, MA), and rinsing with PBS containing 0.1% Triton X-100 for 60 min, each brain section was mounted in 50% glycerol for visualization.

The sections were examined under an inverted ZEISS LSM 7 DUO confocal laser-scanning microscope using a 20× air objective with a numerical aperture of 0.5. All images were obtained under similar conditions (laser intensity and photomultiplier voltage), and usually on the same day. Morphometric analysis of the fluorescently labeled structures was performed offline with Fiji ImageJ (W. Rasband, National Institutes of Health). Areas for the specific antibodies were determined automatically by defining outline masks based on brightness thresholds from maximal projected confocal images [57].

### Data analysis

For power spectral density plots, fast Fourier transformations (FFT, segment length 8192 points) were calculated from 60-s LFP recordings using Axograph X (Kagi, Berkeley, CA). Gamma oscillation power was calculated by integrating power spectral density between 20 and 80 Hz using KaleidaGraph. The shift in the limits for the bandwidth power calculations to 20–80 Hz compared with in vivo (commonly ⁓30–80 Hz) is due to the linear relationship between recording temperature and oscillation frequency [58, 59]. In our experimental set-up, the optimal recording temperature was ⁓6 °C lower than the body temperature, and the bandwidth used for gamma power calculation is a routinely used frequency band in ex vivo recordings of gamma rhythms [52, 55, 59–63]. Thus, in control conditions all the slices included in the study showed a peak frequency between 20 and 80 Hz. In several treatments and in most of the control conditions the spectrograms showed a typical central (peak) frequency accompanied by the corresponding harmonics generated during the FFT. Diverse treatments affected the height of the peak frequency and the harmonics, which accounted for the observed overall reduction of the calculated gamma oscillation power. Peak frequency of gamma oscillations and frequency variance (full width at a half-maximum) were obtained using Axograph X. For further analysis of LFP recordings, the signals were filtered in both directions using a band-pass Butterworth filter set to 20–50 Hz for autocorrelation analysis of gamma oscillations. This band-pass filtering allows analysis of autocorrelations of the major bulk of the signal around the central frequency according to the spectrograms displayed by fast Fourier transformations. Normalized autocorrelations were performed using Matlab custom-written routines. The coefficient of rhythmicity (Cr) was calculated from the autocorrelograms as a measure of the quality of gamma oscillations [51, 62] or EPSC and IPSC rhythmicity [62], and was defined as follows: Cr = (α − β)/(α + β), where (1 + α) and (1 + β) corrections were applied. The value of α corresponds to the value of the height of the second peak and β to the first trough in the autocorrelogram counting the first peak at zero lag. Cr ranges between 0 and 1, with higher coefficient values denoting more rhythmic activity and thus better rhythm quality. Following the previous inclusion criteria for rhythmicity assessment using Cr [51, 62], in the current study all the recordings displayed Cr ≥ 0.01.

FSN and PC spike-phase coupling with corresponding gamma-LFP (60-s long) was analyzed using a custom-made Matlab routine as previously described [51, 52, 62]. LFP traces were filtered using a band-pass Butterworth filter (20–40 Hz, both directions) and APs were detected using an amplitude threshold set to 30%–50% of the total AP amplitude. A narrower band-pass (20–40 Hz) compared to the filtering for autocorrelations allows to evaluate the phase-locking around a narrower frequency-band where the detection of individual cell contribution to the central activity of the network for the gamma rhythm is more precise. The instantaneous phase-angle of gamma oscillations for each AP was determined using a Hilbert transform. Firing window was analyzed by distributing phase-angles of all AP and gamma oscillation-phases (in radians) in polar-plots, with the peak of the oscillation cycle corresponding to 0 π and the trough to π in the polar plots. For each AP, a vector of length = 1 was assigned at the angle corresponding to the phase of the LFP. An averaged resultant phase-density vector was used to describe the preferred phase of firing (phase-angle) and how recurrent the firing in that angle was (vector length). Thus, a larger vector denotes a stronger spike-phase coupling/more synchronized AP firing relative to the network gamma oscillation. Vector length is shown normalized by the total number of AP for each cell recorded and the summary is plotted for each condition. All firing distributions were assessed for uniformity setting the inclusion criteria at *P* < 0.05 with a Rayleigh’s test.

EPSCs and IPSCs were detected off-line using a custom-made template in Clampfit 10.4 software including no less than 20 averaged representative events. EPSC and IPSC parameters (charge transfer, event amplitude and inter-event-interval distributions) were analyzed using GraphPad Prism (GraphPad Software, Boston, MA) with the results representing average values taken over 60-s periods. For the time domain analysis (cross-correlation) the same 60-s segment of EPSCs/IPSCs and their corresponding LFPs were filtered (Butterworth, 20–40 Hz). Normalized cross-correlation index (XC) between EPSCs/IPSCs and the LFP was calculated with a Matlab custom-written routine. The cross-correlation analysis displayed several peaks with periodic fluctuations evidencing the oscillatory nature of the signals. The magnitude and lag of the central negative peak from the XC function were used to describe the similarity and phase-shift of both signals, respectively [62, 64].

### Drugs and chemicals

All chemical compounds used in intracellular and extracellular solutions were obtained from Sigma-Aldrich Sweden AB (Stockholm, Sweden). KA was dissolved in miliQ water. Human gal3 protein was produced by the protein production unit LP3 at Lund University. Briefly, the gal3 protein was produced in *Escherichia coli* strain TUNER(DE3) / pET3c-hum-gal3 grown in LB medium at 18 °C, 250 rpm with 1 mM IPTG overnight. After cell lysis and ultracentrifugation, gal3 was purified on a 20-ml lactocyl-sepharose column. Peak fractions containing gal3 were pooled and dialyzed against PBS, pH 7.4. Human gal3 was analyzed on a Criterion TGX AnykD precast SDS-PAGE gel (Bio-Rad) stained with Bio-Safe Coomassie (Bio-Rad). The purity was estimated to be > 95%. The inhibitor TD139 (1,1′-sulfanediyl-bis-3-deoxy-3-4-3-fluorophenyl-1 H-1,2,3-triazol-1-yl-β-d-galactopyranoside) was acquired from Chemtronica (Stockholm, Sweden) and dissolved in DMSO to a final dilution of ≤ 0.001% of DMSO when applied to brain slices.

### Statistical analysis

All statistical analysis was performed using GraphPad Prism 8.0 either in absolute values or normalized data (to 5 min of stable baseline) when appropriate. To minimize the variation between slices, a paired statistical design was used when compounds were washed-in sequentially and unpaired when treatments were performed independently of each other. Two- or one-sided designs were used as appropriate. Prior to statistical analysis, all data were subjected to detection of outliers, which were removed using the ROUT method. This was followed by tests for normality distribution and variance similarity between groups. Wilcoxon’s signed rank sum test was used when reporting statistical differences between control conditions and compound effects in the non-parametrically distributed data. A paired Student’s *t* test was used when the data were distributed normally. For multiple comparisons, ordinary one-way ANOVA followed by Holm-Sidak’s multiple comparisons test or Kruskal-Wallis test followed by Dunn’s multiple comparisons was carried out depending on the parametric or non-parametric nature of the data, respectively. Results are reported as mean ± SEM and significance levels were set as follows: *P < 0.05, **P < 0.01, ***P < 0.005, ****P < 0.0001. ‘*N*’ means number of animals and ‘*n*’ means number of slices or cells depending on the study configuration.

## Results

### Gal3 induces degradation of gamma oscillations via its CRD

**Fig. 1:**
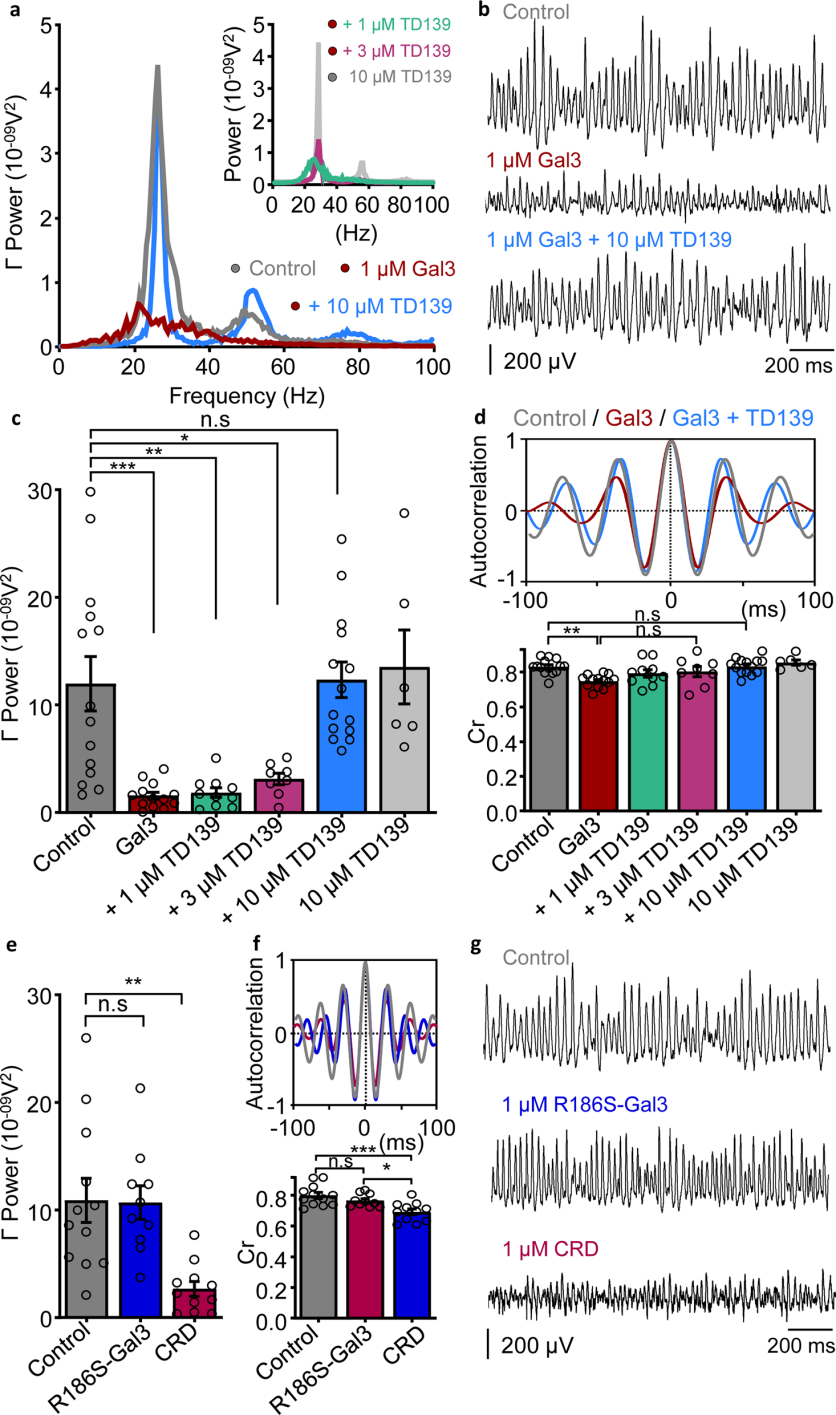
Gal3 impairs gamma oscillation power and rhythmicity through its carbohydrate-recognition domain (CRD). **a** Representative power spectra of hippocampal CA3 network activity recorded in control slices (gray), slices pre-incubated for 15 min with 1 µM gal3 (red) and slices co-incubated with 1 µM gal3 + 10 µM TD139 (blue). Inset: Representative power spectra for slices co-incubated with 1 µM gal3 + 10 µM TD139 (gray), co-incubated with 1 µM gal3 + 3 µM TD139 (magenta) or co-incubated with 1 µM gal3 + 1 µM TD139 (green). **b** Representative example traces of recordings performed in the conditions shown in **a**. **c** Summary bar graphs of gamma oscillation power for the conditions shown in **a**, demonstrating that 10 µM TD139 confers the most effective prevention against gal3-induced decrease of gamma oscillation power (ordinary one-way ANOVA followed by Holm-Sidak’s multiple comparisons test, Additional file 2: Table S1): control (gray, 12.0 ± 2.52 × 10^–09^ V^2^, *n* = 14, *N* = 5); gal3 (red, 1.60 ± 0.33 × 10^–09^ V^2^, *n* = 13, *N* = 3); gal3 + 1 µM TD139 (green, 1.88 ± 0.45 × 10^–09^ V^2^, *n* = 10, *N* = 3); gal3 + 3 µM TD139 (magenta, 3.13 ± 0.53 × 10^–09^ V^2^, *n* = 8, *N* = 4); gal3 + 10 µM TD139 (blue, 12.3 ± 1.63 × 10^–09^ V^2^, *n* = 14, *N* = 3). 10 µM TD139 applied alone did not affect gamma oscillations power (light gray, 13.5 ± 3.44 × 10^–09^ V^2^, *n* = 6, *N* = 2). **d** Coefficient of rhythmicity calculated from the autocorrelation function as a measure of gamma oscillation quality (see methods). Top: Representative autocorrelation of gamma oscillations recorded in control conditions (gray), in slices pre-incubated with gal3 (red) and slices co-incubated with gal3 + 10 µM TD139 (blue). Bottom: Bar graphs summarizing the Cr calculated for each condition listed in **c**: control (gray, 0.83 ± 0.01, *n* = 14, *N* = 5); gal3 (red, 0.75 ± 0.01, *n* = 13, *N* = 3); gal3 + 1 µM TD139 (green, 0.79 ± 0.02, *n* = 10, *N* = 3); gal3 + 3 µM TD139 (magenta, 0.8 ± 0.03, *n* = 8, *N* = 4); gal3 + 10 µM TD139 (blue, 0.83 ± 0.01, *n* = 14, *N* = 3). 10 µM TD139 applied alone did not affect gamma oscillation rhythmicity (light gray, 0.85 ± 0.02, *n* = 6, *N* = 2). Statistical testing performed by ordinary one-way ANOVA followed by Holm-Sidak’s multiple comparisons test (Additional file 2: Table S2). **e** Bar graph summary of gamma oscillation power from slices recorded in control conditions (gray, 10.9 ± 2.04 × 10^–09^ V^2^, *n* = 12, *N* = 6), slices pre-incubated for 15 min with 1 µM R186S-Gal3 (blue, 10.7 ± 1.56 × 10^–09^ V^2^, *n* = 10, *N* = 3;* P* = 0.9265 vs control) and slices pre-incubated for 15 min with 1 µM CRD-Gal3 (red, 2.67 ± 0.69 × 10^–09^ V^2^, *n* = 11, *N* = 3;* P* = 0.0022 vs control,* P* = 0.0029 vs R186S-Gal3), ordinary one-way ANOVA followed by Holm-Sidak’s multiple comparisons test. **f** Top: Representative autocorrelations of gamma oscillations recorded in the conditions mentioned in **e**. Bottom: Summary bar graphs of the Cr measured in control conditions (gray, 0.8 ± 0.02, *n* = 12, *N* = 6); slices pre-incubated with R186S-Gal3 (blue, 0.76 ± 0.01, *n* = 10, *N* = 3;* P* = 0.1866 vs control), and slices pre-incubated with Gal3-CRD (red, 0.69 ± 0.02, *n* = 11, *N* = 3;* P* = 0.0004 vs control,* P* = 0.0159 vs R186S-Gal3), ordinary one-way ANOVA followed by Holm-Sidak’s multiple comparisons test. **g** Representative example traces of recordings performed in the conditions shown in **e** and **f**. Data are presented as mean ± SE. Significance levels are shown as *P < 0.05, **P < 0.01, ***P < 0.001. n.s: no significant statistical difference; *n*: number of slices; *N*: number of animals

We first evaluated the effect of gal3 on gamma oscillation power and rhythmicity. For this purpose, hippocampal slices of WT mice were incubated for 15 min with 1 µM gal3 and transferred to an interface-type recording chamber. Gamma oscillations were elicited with 100 nM KA and allowed to stabilize for 30 min, following which gamma-local field potential (gamma-LFP) was recorded and analyzed [51, 53]. Incubation with gal3 prior to gamma oscillation induction resulted in a drastic decrease of gamma oscillation power (Fig. 1c, Additional file 2: Table S1) and rhythmicity (Fig. 1d, Additional file 2: Table S2), as observed in the Cr analysis used as an indicator of gamma oscillation quality (see Methods). Gal3 also decreased the gamma power of cholinergic-induced gamma oscillations (Additional file 1: Fig. S2). Co-incubation of slices with gal3 and 1 or 3 µM of gal3 inhibitor TD139 prior to gamma oscillation induction did not prevent the gal3-induced decrease of gamma oscillation power, while 10 µM TD139 conferred an efficient protection (Fig. 1c, Additional file 2: Table S1). Treatment with 10 µM TD139 alone did not exert additional changes compared to control gamma power (Fig. 1c, Additional file 2: Table S1). Interestingly, Cr analysis revealed that 1 and 3 µM TD139 partially prevented gal3-induced disruption of gamma rhythmicity and 10 µM TD139 fully counteracted this gal3-mediated loss of gamma quality (Fig. 1d, Additional file 2: Table S2). As for the power analysis above, incubation of the neuronal network with 10 µM TD139 alone did not change gamma oscillation rhythmicity (Fig. 1d, Additional file 2: Table S2).

Prominent cellular actions of gal3 have been reported to be mediated by its characteristic CRD [27, 33]. Thus, we investigated whether the deleterious effects of gal3 on network oscillations were driven by its CRD. For this purpose, hippocampal slices of WT mice were incubated for 15 min with 1 µM of gal3-isolated CRD as well as with 1 µM of R186S-Gal3, and then transferred to an interface-type recording chamber. R186S-Gal3 is a mutated version of gal3 with reduced affinity for many glycoproteins and for the disaccharide LacNAc, which is the most common minimal galectin-binding moiety in glycoproteins [65, 66]. Recordings performed 30 min after the induction of gamma oscillations revealed that the effects of gal3-CRD paralleled those of intact gal3: decrease of gamma power (Fig. 1e) and impairment of gamma oscillation rhythmicity (Fig. 1f). Notably, R186S-Gal3 did not affect either gamma power (Fig. 1e) or rhythmicity (Fig. 1f). Thus, gal3 induces a drastic degradation of gamma oscillations in the hippocampal network through its CRD and this effect is prevented with highest efficacy by 10 µM TD139.

### Gal3 disrupts action potential phase-lock of fast-spiking interneurons to gamma oscillations in a concentration- and exposure time-dependent manner

**Fig. 2:**
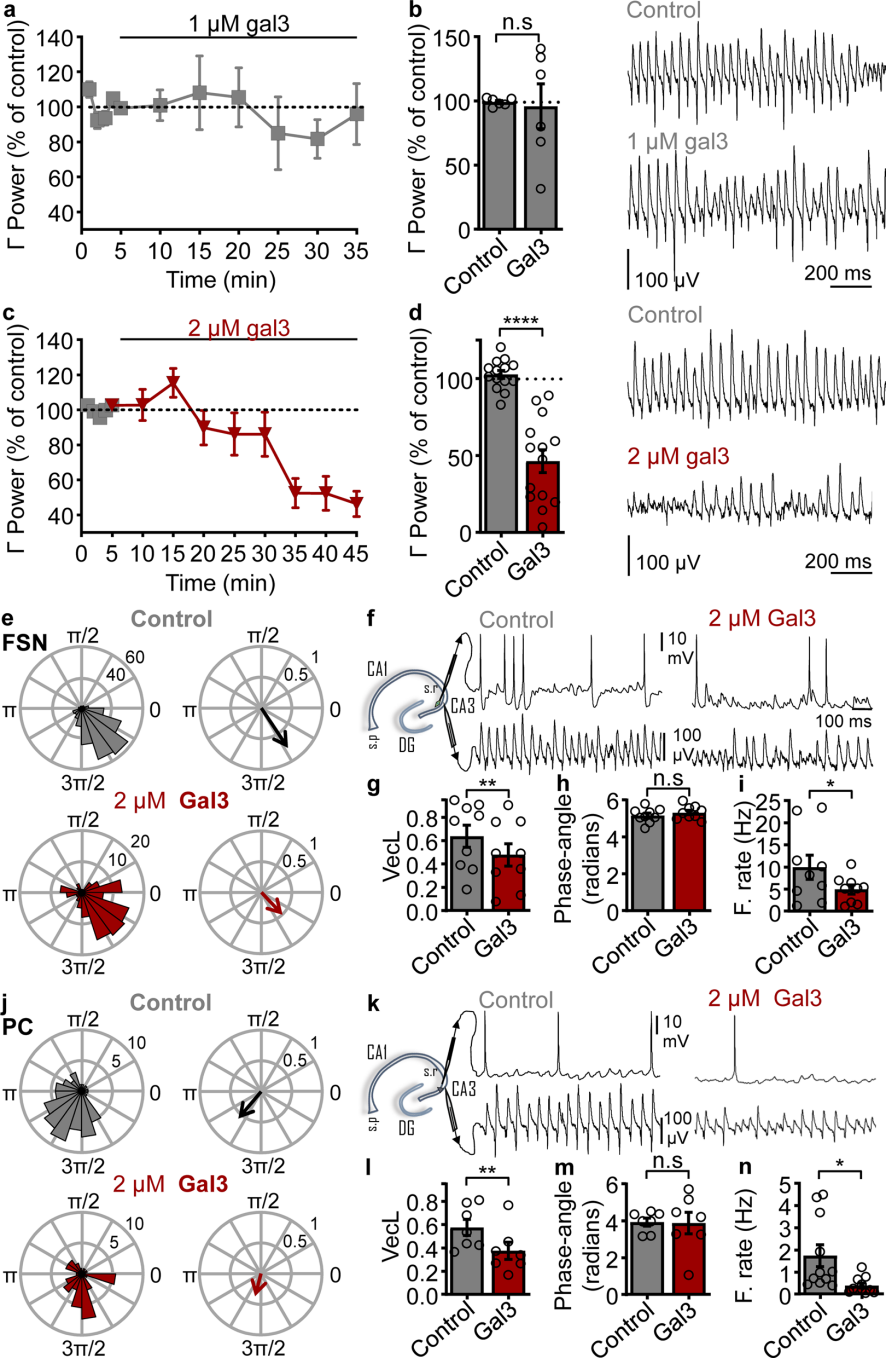
Gal3-induced impairment of FSN and PC action potential phase lock to gamma oscillations. **a** Time course of the effect of 1 µM gal3 application on gamma oscillation power. **b** Left: Summary of gamma power in control condition (99.2% ± 1.25%) and 30 min after 1 µM gal3 application (95.8% ± 17.5%, *n* = 6, *N* = 3; *P* > 0.9999 vs control, two-tailed Wilcoxon test). Right: Representative example traces of the conditions shown in the bar graph. **c** Time course of the effect of 2 µM gal3 application on gamma oscillation power. **d** Left: Summary of gamma power in control condition (gray, 102.6% ± 2.6%) and 40 min after 2 µM gal3 application (46.4% ± 7.39%, *n* = 14, *N* = 7; *P* > 0.9999, two-tailed Wilcoxon test). Right: Representative example traces of the conditions shown in the bar graph. **e** Representative polar plots of the AP firing window for a FSN recorded concomitantly to gamma oscillations in control conditions (gray) and 40 min after application of 2 µM gal3 (red). Left: FSN-AP firing window relative to gamma. Right: resultant vector showing the magnitude of the phase-lock and the gamma phase-angle preference. **f** Representative example traces of FSN AP firing (upper traces) concomitantly recorded with gamma oscillations (lower traces) in conditions mentioned in **e** and **d**. Left: schematic of the hippocampus showing the locations of FSN AP and LFP recordings. **g** Quantification of the vector length (control: 0.64 ± 0.1, gal3: 0.48 ± 0.1, *n* = 9, *N* = 6;* P* = 0.0092, two-tailed *t*-test). **h** Quantification of the gamma-preferred phase-angle (control: 5.2 ± 0.13 radians, gal3: 5.3 ± 0.13 radians, *n* = 9, *N* = 6;* P* = 0.1459, two-tailed *t*-test). **i** Quantification of the FSN firing rate (control: 9.91 ± 2.73 Hz, gal3: 4.9 ± 1, *n* = 9, *N* = 6;* P* = 0.0389, two-tailed* t*-test). **j** Representative polar plots of the AP firing window for a PC recorded concomitantly to gamma oscillations in control conditions (gray) and 40 min after application of 2 µM gal3 (red). Left: PC AP firing window relative to gamma. Right: resultant vector showing the magnitude of the phase-lock and the gamma phase-angle preference. **k** Representative example traces of PC AP firing (upper traces) concomitantly recorded with gamma oscillations (lower traces) in conditions mentioned in **j** and **k**. **l** Quantification of the vector length (control: 0.58 ± 0.1, gal3: 0.38 ± 0.1, *n* = 7, *N* = 6;* P* = 0.0078, one-tailed Wilcoxon test). **m** Quantification of the gamma-preferred phase-angle (control: 3.9 ± 0.21, gal3: 3.9 ± 0.58, *n* = 7, *N* = 6;* P* = 0.5781, two-tailed Wilcoxon test). **n** Quantification of the PC firing rate (control: 1.74 ± 0.5 Hz, gal3: 0.38 ± 0.11, *n* = 11, *N* = 6;* P* = 0.0389, two-tailed paired *t*-test). Data are presented as a mean ± SE. Significance levels are shown as *P < 0.05, **P < 0.01, ***P < 0.001. n.s: no significant statistical difference; *n*: number of slices recorded in **a**–**d** and cells recorded in **e**–**n**; *N*: number of animals

We evaluated the effect of gal3 on fast-spiking interneuron phase-lock to ongoing gamma oscillations. To this end, we performed concomitant recordings of LFP and whole-cell patch-clamp recordings of FSN activity in a submerged-type recording chamber during the activated network state (gamma oscillations). Thirty minutes after gamma oscillation induction, 5 min of control gamma and FSN firing were recorded and subsequently gal3 was bath-applied. Application of 1 µM gal3 for 30 min did not induce a decrease of gamma oscillation power (Fig. 2a, b), indicating that its effect may depend on the activation state of the network. Likewise, FSN firing phase-lock and AP firing rate were not affected by the application of 1 µM gal3 to the activated network (Additional file 1: Fig. S3).

Using the same approach of submerged-type recording chamber and gal3 application to the activated network, we increased gal3 concentration and the time of application. 2 µM gal3 gradually decreased gamma oscillation power starting 15 min after application and reaching a significant stable reduction at 40 min after wash-in (Fig. 2c, d). Concomitantly, FSN-gamma phase-lock was severely disrupted (Fig. 2e–i), evidenced by a significant decrease of the resultant vector length of the AP firing distribution within gamma phases (Fig. 2e, g). The preferred gamma phase-angle was not affected (Fig. 2h), whereas phase-lock impairment was accompanied by a drastic decrease of FSN firing rate (Fig. 2e, f, i).

During ongoing gamma oscillations, FSN pace the activity of PCs [7–9, 54]. Thus, any alterations of FSN activity within the network could affect PC activity during ongoing gamma oscillations. To further address this possible causal link to PCs, we performed the same experiment used for assessing FSN (Fig. 2e–i) in PCs. Similar to the FSN experiment, 40-min application of 2 µM gal3 during the ongoing gamma oscillations resulted in a drastic desynchronization of PC AP firing (Fig. 2j–n). This was evidenced by a widening of the firing window, which resulted in a shorter vector length (Fig. 2j, l) that was accompanied by a drastic decrease of AP firing rate (Fig. 2j, k, n). The preferred phase-angle of AP firing was not affected by gal3 wash-in (Fig. 2m).

### Gal3 impairs inhibitory and excitatory synaptic transmission during ongoing gamma oscillations

**Fig. 3:**
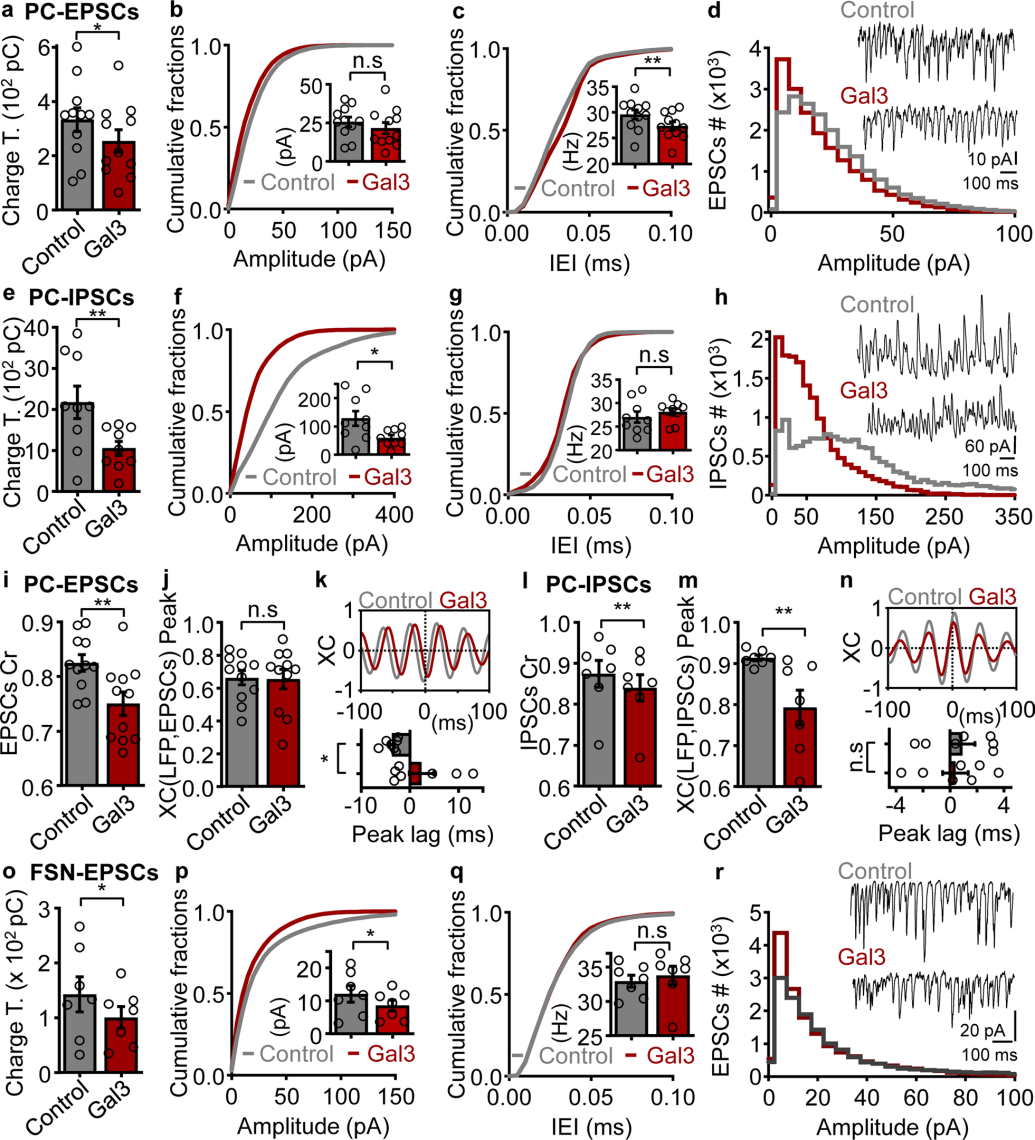
Gal3-induced impairment of excitatory and inhibitory synaptic transmission during gamma oscillations.** a** Summary bar graphs of EPSC charge transfer in PCs in control conditions (gray) and 40 min after application of 2 µM gal3 (red) (control: 333.2 ± 44.4 pC, gal3: 253.9 ± 41.3 pC, *n* = 11, *N* = 6;* P* = 0.0423, one-tailed paired *t*-test). **b** Cumulative probability of PC EPSC amplitude in control conditions (gray) and 40 min after application of 2 µM gal3 (red). Inset: Quantification of mean amplitude (control: 25.7 ± 3.1 pA, gal3: 21.5 ± 3.68 pA, *n* = 11, *N* = 6;* P* = 0.2158, two-tailed paired *t*-test). **c** Cumulative probability of PC EPSC frequency in control conditions (gray) and 40 min after application of 2 µM gal3 (red). Inset: Quantification of mean frequency (control: 29.6 ± 0.91 Hz, gal3: 27.4 ± 0.79 Hz, *n* = 11, *N* = 6;* P* = 0.0046, two-tailed paired *t*-test). **d** EPSC amplitude distribution in conditions mentioned in **a**. Inset: Representative traces of EPSC recordings for each condition. **e** Summary bar graphs of IPSC charge transfer in PCs in control conditions (gray) and 40 min after application of 2 µM gal3 (red) (control: 2170 ± 395.4 pC, gal3: 1052 ± 171.3 pC, *n* = 9, *N* = 4;* P* = 0.0049, one-tailed paired *t*-test). **f** Cumulative probability of PC IPSC amplitude in control conditions (gray) and 40 min after application of 2 µM gal3 (red). Inset: Quantification of mean amplitude (control: 128.8 ± 26.3 pA, gal3: 59.5 ± 9.72 pA, *n* = 9, *N* = 4;* P* = 0.0143, two-tailed paired* t*-test). **g** Cumulative probability of PC IPSC frequency in control conditions (gray) and 40 min after 2 µM gal3 application (red). Inset: Quantification of mean frequency (control: 27.0 ± 1.17 Hz, gal3: 28.1 ± 0.74 Hz, *n* = 9, *N* = 4;* P* = 0.2004, two-tailed paired *t*-test). **h** IPSC amplitude distribution in conditions mentioned in **e**. Inset: Representative traces of IPSC recordings for each condition. **i** Coefficient of rhythmicity calculated from PC EPSCs in the conditions described in **a** (control: 0.82 ± 0.02, gal3: 0.75 ± 0.02, *n* = 11, *N* = 6;* P* = 0.0040, two-tailed paired *t*-test). **j** Analysis of the cross-correlation (XC) (LFP, EPSCs) peak size revealed that gal3 did not alter the LFP-EPSC similarity (control: 0.66 ± 0.04, gal3: 0.65 ± 0.06, *n* = 11, *N* = 6;* P* = 0.8994, two-tailed Wilcoxon test). **k** Top: Representative XC calculation performed between gamma LFP and EPSC signals in the conditions described in **a**. Bottom: Analysis of the peak lag of the XC (LFP, EPSCs) revealed that gal3 induced a phase shift in the maximal coordination between LFP and EPSCs (control: − 3.71 ± 0.54 ms, gal3: 2.43 ± 2.53, *n* = 7, *N* = 6;* P* = 0.0156, two-tailed Wilcoxon test). **l** Coefficient of rhythmicity calculated from PC IPSCs in the conditions described in **e,** showing that gal3 drastically affected IPSC rhythmicity (control: 0.87 ± 0.03, gal3: 0.84 ± 0.03, *n* = 7, *N* = 4;* P* = 0.0078, one-tailed Wilcoxon test). **m** Quantification of the XC (LFP, IPSCs) peak size showing that gal3 impaired LFP-IPSC similarity (control: 0.91 ± 0.01, gal3: 0.79 ± 0.04, *n* = 7, *N* = 4;* P* = 0.0078, one-tailed Wilcoxon test). **n** Top: Representative XC calculation performed between gamma LFP and IPSC signals. Bottom: Analysis of the peak lag of the XC (LFP, IPSCs) revealed that gal3 induced a non-significant phase shift in the maximal coordination between LFP and IPSCs (control: 0.91 ± 0.91 ms, gal3: 0.4 ± 0.94, *n* = 7, *N* = 4;* P* = 0.2969, two-tailed Wilcoxon test). **o** Quantification of the excitatory charge transfer (control: 142.8 ± 31.8 pC, gal3: 100.7 ± 19.6 pC, *n* = 7, *N* = 4;* P* = 0.0146, one-tailed Wilcoxon test). **p** Cumulative probability of FSN EPSC amplitude in control conditions (gray) and 40 min after application of 2 µM gal3 (red). Inset: Quantification of mean amplitude (control: 12.1 ± 2.5 pA, gal3: 8.59 ± 1.63 pA, *n* = 7, *N* = 4;* P* = 0.0313, two-tailed Wilcoxon test). **q** Cumulative probability of FSN EPSC frequency in control conditions (gray) and 40 min after application of 2 µM gal3 (red). Inset: Quantification of mean frequency (control: 33.0 ± 0.89 Hz, gal3: 33.8 ± 1.35 Hz, *n* = 7, *N* = 4;* P* = 0.6875, two-tailed Wilcoxon test). **r** FSN EPSC amplitude distribution in the conditions mentioned in **o**. Inset: Representative traces of EPSC recordings for each condition. Data are presented as mean ± SE. Significance levels are shown as *P < 0.05, **P < 0.01. IEI: Inter-event interval; n.s: no significant statistical difference; *n*: number of slices and cells recorded concomitantly; *N*: number of animals

Proper coordination of excitatory and inhibitory synaptic activities within neuronal networks is crucial for circuit entrainment and emergence of brain rhythms [9, 12, 54, 67, 68]. In the above experiments, we observed that gal3 impaired the firing coordination relative to gamma oscillations of crucial neuronal populations such as inhibitory GABAergic FSN and excitatory glutamatergic PCs. We proceeded on to investigate the effect of gal3 on the excitatory and inhibitory synaptic inputs onto PCs as well as excitatory drive onto FSN. For this purpose, we recorded excitatory and inhibitory postsynaptic currents (EPSCs and IPSCs, respectively) from PCs (see Methods) 40 min after application of 2 µM gal3 during ongoing gamma oscillations (Fig. 3).

Analysis of EPSC charge transfer showed that gal3 significantly decreased the overall excitatory input onto PCs (Fig. 3a), which was accompanied by a decrease of EPSC frequency (Fig. 3c). At the same time, gal3 caused a shift of the EPSC amplitude distribution towards smaller components (Fig. 3d), while the mean amplitude remained unaffected (Fig. 3b). Additionally, gal3 impaired both the EPSC rhythmicity within the gamma frequency-band and the PC-EPSC to gamma-LFP phase relationship (Fig. 3i–k).

Interestingly, inhibitory drive onto PCs also showed a decrease 40 min after gal3 application. This was evidenced by a significant decrease of the overall IPSC charge transfer (Fig. 3e) caused by a decrease of IPSC amplitude (Fig. 3f). In contrast, IPSC frequency remained unchanged after gal3 wash-in (Fig. 3g). Gal3 also shifted the IPSC amplitude distribution, decreasing larger-amplitude components while inducing an increase of smaller-amplitude components (Fig. 3h). Similar to EPSCs, gal3 affected IPSC rhythmicity and, notably, the PC-IPSC to gamma-LFP phase relationship was weakened (Fig. 3l–n).

Gal3 also decreased the overall excitatory input onto FSN as evidenced by a significant decrease of EPSC charge transfer (Fig. 3o), which was accompanied by a decrease of EPSC amplitude (Fig. 3p) and increased occurrence of small-amplitude components (Fig. 3r). However, the overall mean FSN-EPSC frequency was not affected (Fig. 3q). Notably, gal3 also disrupted EPSC rhythmicity in FSN (Additional file 1: Fig. S4a) as well as the relationship between EPSCs and LFP within the gamma frequency-band (Additional file 1: Fig. S4b). These findings show that gal3 dramatically impairs overall excitatory and inhibitory inputs onto PCs. This is accompanied by a loss of excitation and inhibition rhythmicity, and an impairment of the relationship between postsynaptic currents and the gamma rhythm, a finding that shares commonalities with the observed gal3-induced impairment of excitatory drive onto FSN.

### Neuronal mechanisms underlying the activity-dependence of gal3-induced impairment of functional network dynamics

**Fig. 4:**
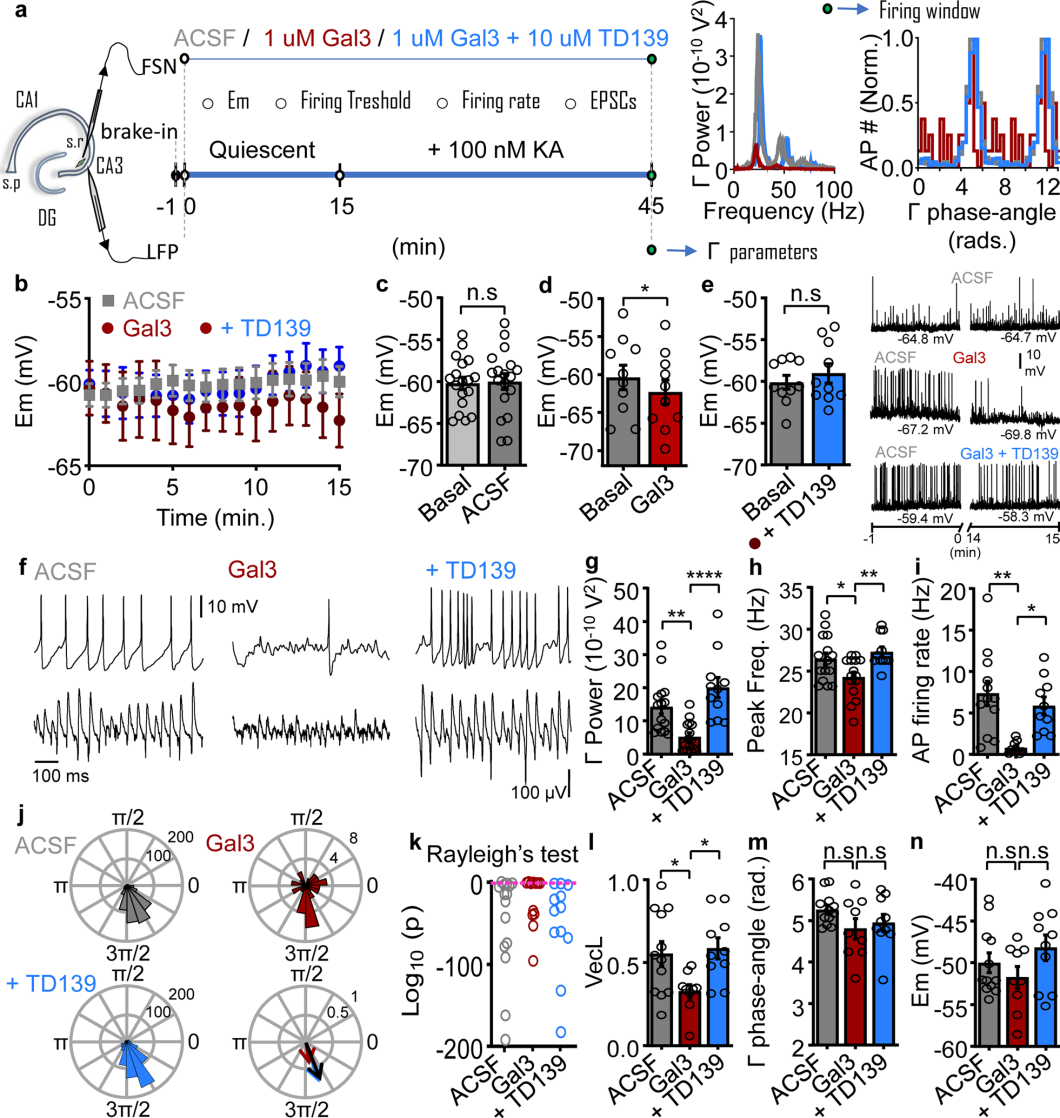
Neuronal mechanisms underlying the activity-dependence of gal3-induced impairment of gamma oscillations. **a** Left, general schematic representation of the experimental set up. Right, representative power spectra of network activity (0–100 Hz) and representative FSN AP firing windows relative to concomitant gamma oscillation 30 min after KA application for the color-coded experimental conditions shown on the left. **b**–**e** correspond to recordings of FSN membrane potential performed in the quiescent network state for 15 min. Additional measurements for each condition (control, gal3 or gal3 + TD139 application) such as firing at basal membrane potential and firing threshold are provided in Fig. S3. Effects on EPSCs in basal state are provided in Additional file 1: Fig. S4. **f**–**n** correspond to FSN-gamma phase-lock analyzed from concomitant recordings of the same FSN and gamma network activity for 30 min after 100 nM KA application to induce stable gamma oscillations. Additional measurements for each condition in the activated state such as FSN EPSCs are provided in Additional file 1: Fig. S5. Note that both recording electrodes (whole cell FSN patch clamp and LFP recording) were positioned and left in place for the entire experiment (left hippocampal diagram), including gal3 or gal3 + TD139 applications during the quiescent state recordings (first 15 min) and during the subsequent gamma induction (following 30 min). **b** Time course of the effect of 15 min wash-in of 1 µM gal3 (red) or co-application of 1 µM gal3 + 10 µM TD139 (blue) on FSN membrane potential (Em) in the quiescent network state. Basal control condition (ACSF) is shown in gray. **c**–**e** show the quantification of FSN membrane potential over 15 min of control recordings (basal: − 60.2 ± 0.73 mV, ACSF: − 60.0 ± 0.92 mV, *n* = 18, *N* = 6;* P* = 0.7427), after gal3 (basal: − 60.4 ± 1.61 mV, gal3: − 62.3 ± 1.60 mV, *n* = 10, *N* = 7;* P* = 0.0346) or after gal3 + TD139 (basal: − 60.1 ± 0.82 mV, gal3: − 59.0 ± 1.14 mV, *n* = 10, *N* = 5;* P* = 0.1804). Statistics performed: two-tailed paired *t*-test. Inset: Example traces showing 1 min recorded in basal (control) condition and the last minute recorded for the quantification of the effect of gal3 (red), gal3 + TD139 (blue) as well as the control recorded just with ACSF (gray). Corresponding effects on AP firing for each condition when the FSN was firing at basal membrane potential are shown in Additional file 1: Fig. S5. **f** Representative traces of concomitant recordings (upper: APs, lower: gamma oscillations) in control conditions as well as in the presence of gal3 or gal3 + TD139. **g** Summary of gamma oscillation power calculated from 20 to 80 Hz for each condition showing that co-application of TD139 counteracts the gal3-induced decrease of gamma oscillation power (ACSF: 1.43 ± 0.21 × 10^–09^ V^2^, *n* = 15, *N* = 6; gal3: 0.52 ± 0.12 × 10^–09^ V^2^, *n* = 15, *N* = 7;* P* = 0.0050 vs control, *P* < 0.0001 vs gal3 + TD139, gal3 + TD139: 2 ± 0.31 × 10^–09^ V^2^, *n* = 11, *N* = 5;* P* = 0.0644 vs control). **h** Summary of gamma peak frequency showing that co-application of TD139 counteracts the gal3-induced slowing of the gamma rhythm (ACSF: 26.5 ± 0.71 Hz, *n* = 15, *N* = 6; gal3: 24.3 ± 0.63 Hz, *n* = 15, *N* = 7;* P* = 0.0372 vs control, *P* < 0.0099 vs gal3 + TD139, gal3 + TD139: 27.3 ± 0.59 Hz, *n* = 11, *N* = 5;* P* = 0.3867 vs control). **i** Summary of AP firing rate showing that gal3-induced decrease of AP rate is prevented by co-application of TD139 (ACSF: 7.36 ± 1.47 Hz, *n* = 12, *N* = 6; gal3: 0.79 ± 0.26 Hz, *n* = 9, *N* = 7;* P* = 0.0012 vs control, *P* < 0.0118 vs gal3 + TD139, gal3 + TD139: 5.88 ± 1 Hz, *n* = 10, *N* = 5;* P* = 0.3619 vs control). **j** Representative polar plots of the firing windows shown in **a** for each condition with the resultant vector (bottom right) showing the magnitude of the FSN-gamma phase-lock and the phase-angle preference for each condition. **k** Logarithmic distribution of the *P* values from the Rayleigh’s test for uniformity showing that in the presence of gal3 considerably fewer FSN are able to engage in a patterned firing locked to a specific gamma phase (ACSF: 1 out of 15 recorded cells, gal3: 3 out of 12 recorded cells, gal3 + TD139: all the recorded cells showed *P* < 0.05). Pink dashed line denotes* P* = 0.05. **l** Summary of the resultant vector length for each condition (ACSF: 0.55 ± 0.07, *n* = 12, *N* = 6; gal3: 0.33 ± 0.04, *n* = 9, *N* = 7;* P* = 0.0364 vs control, *P* < 0.0308 vs gal3 + TD139, gal3 + TD139: 0.59 ± 0.06, *n* = 10, *N* = 5;* P* = 0.7143 vs control). **m** Quantification of the phase-angle firing preference revealing that neither gal3 nor gal3 + TD139 induced significant changes (ACSF: 5.26 ± 0.11 radians, *n* = 12, *N* = 6; gal3: 4.81 ± 0.24 radians, *n* = 9, *N* = 7;* P* = 0.2328 vs control,* P* = 0.5881vs gal3 + TD139, gal3 + TD139: 5 ± 0.2 radians, *n* = 10, *N* = 5;* P* = 0.3921 vs control). **n** Summary bar graphs showing that 30 min of KA application did not differentially depolarize FSN either in control conditions, or in the presence of gal3 or gal3 + TD139 (ACSF: − 50 ± 1.17 mV, *n* = 12, *N* = 6; gal3: − 51.8 ± 1.32 mV, *n* = 9, *N* = 7;* P* = 0.5595 vs control,* P* = 0.2304 vs gal3 + TD139, gal3 + TD139: − 48.2 ± 1.54 mV, *n* = 10, *N* = 5;* P* = 0.5595 vs control). Data are presented as mean ± SE. Statistics performed: ordinary one-way ANOVA followed by Holm-Sidak’s multiple comparisons test. Significance levels are shown as *P < 0.05, **P < 0.01, ****P < 0.0001. n.s: no significant statistical difference; *n*: number of cells recorded in **b**–**e**, number of slices recorded in **g**–**h** and number of cells and slices recorded concomitantly in **i**, **k**–**n**; *N*: number of animals

Here we observed significant reductions of gamma power and rhythmicity when slices were exposed to gal3 (1 µM) prior to gamma induction (Fig. 1); however, when the network is under stable entrainment into the gamma rhythm (30 min after gamma induction by 100 nM KA), an increased concentration of gal3 is needed to observe comparable disruption (Fig. 2). To investigate the effects of gal3 on FSN basal activity and its possible consequences for the network function during gamma oscillations, we performed recordings of FSN activity concomitantly with LFP, first in the basal state and then during subsequent gamma oscillation induction in CA3 region of hippocampal WT mouse slices (Fig. 4a). Gal3 or gal3 plus gal3 inhibitor (TD139) was applied for 15 min prior to gamma oscillation induction and remained in the bath solution throughout the subsequent 30 min of rhythm establishment and stabilization. Basal state recordings also served as a control to ensure that neither the whole-cell patch-clamp configuration nor the bath ACSF induced functional deviations during the total 45-min-long recordings (Fig. 4a).

Recordings of FSN membrane potential in the quiescent state revealed that gal3 hyperpolarized FSN membrane potential 15 min after bath application (Fig. 4b, d). Co-application of gal3 and TD139 prevented this gradual hyperpolarization (Fig. 4b, e), which was also absent in the control condition (ACSF, Fig. 4b, c). In parallel to the observed hyperpolarization of FSN membrane potential, gal3 induced an increase of the FSN AP firing threshold (Additional file 1: Fig. S5b) that was prevented by co-application of TD139 (Additional file 1: Fig. S5c) and absent in the control condition (ACSF, Additional file 1: Fig. S5a). Interestingly, we found that some FSNs were spontaneously firing APs at basal membrane potential without significant changes of their firing rate during the 15 min of quiescent state recording (Fig. 4e and Additional file 1: Fig. S5c).

We also observed a drastic decrease of the firing rate at basal state following gal3 wash-in (Fig. 4e, inset and Additional file 1: Fig. S5e), probably due to the gal3-induced hyperpolarization of FSN membrane potential and the increase of the firing threshold. The decrease of the firing rate was prevented by co-application of TD139 (Fig. 4e and Additional file 1: Fig. S5f). At the network level, gal3 provoked a decrease of frequency of EPSCs onto FSNs, which was prevented by co-application of TD139 (Additional file 1: Fig. S6d–f). Thus, our findings indicate that 1µM of gal3 in the quiescent state causes hypoactivity in FSN prior to gamma oscillation induction, which then overrides that hypoactivity to a limited extent. This is evident in the fact that concomitant LFP recordings revealed that the neuronal network failed to generate gamma oscillation power similar to that seen in control conditions (Fig. 4f, g). Such gal3-induced impairment of rhythmic network activity was counteracted by the presence of TD139 (Fig. 4f, g). Additionally, gamma oscillations induced in slices previously exposed to gal3, showed lower peak frequency compared to gamma oscillations recorded in control conditions or in the presence of the gal3 inhibitor (Fig. 4f, h).

Treating slices with gal3 led to a drastic decrease of FSN AP firing rate during ongoing gamma oscillations (Fig. 4f, i) [52, 54, 62, 69]. By contrast, FSNs recorded under gal3 inhibition displayed similar firing rates to FSNs recorded in control conditions (Fig. 4f, i). Notably, gal3 significantly prevented FSN spike-phase locking to gamma LFP, evidenced by a drastic decrease of the resultant vector length (Fig. 4j). This was accompanied by an increase of the ratio of FSN that did not show patterned activity relative to the ongoing gamma rhythm (Fig. 4k). Gal3 also led to decreased EPSC frequency and disrupted EPSC rhythmicity onto FSN (Additional file 1: Fig. S7b, d). These functional deviations were prevented in the presence of TD139. In addition, analysis of FSN membrane potential in the activated network state (gamma oscillations) revealed that the gal3-driven decrease of FSN firing rate during gamma oscillations was not caused by the previous hyperpolarization observed in the quiescent state. KA-induced depolarization during gamma oscillation induction resulted in a similar FSN membrane potential in the three studied conditions: (1) control, (2) gal3, and (3) gal3 + TD139 (Fig. 4n).

To investigate a possible effect of gal3 on some of the gal3-activation/responses-associated genes related to microglial AD phenotype, we assessed the expression of *Trem2, Tlr4, Clec7a*, and *Cx3cr1* transcripts in some of the recorded slices (30 min after gamma oscillation induction, see Fig. 4a). Notably, there was no change of expression of *Trem2, Clec7a* or *Tlr4* in slices treated with gal3 prior to gamma oscillation induction (Additional file 1: Fig. S8). Conversely, there was a decrease of the ∆Ct of the astrocyte marker *Gfap* in slices treated with gal3 in the presence of the inhibitor TD139 compared with control slices. KA induced an increase of ∆Ct of the chemokine receptor *Cx3cr1* compared with gal3 or gal3 plus TD139 treatment (Additional file 1: Fig. S8).

### Interference with gal3 signaling prevents the disruption of gamma oscillations in two different AD models

Acute application of physiological concentrations of Aβ42 (50 nM) to hippocampal slices causes a dramatic and rapid impairment of gamma oscillations [51–53, 55, 70, 71]. Also, cognition-relevant brain rhythms have been reported to be disturbed in several AD mouse models [25, 72–76]. We investigated whether inhibition of gal3 signaling in two AD-related mouse models can prevent the disruption of gamma oscillations, which is linked to the cognitive impairment in AD.

**Fig. 5:**
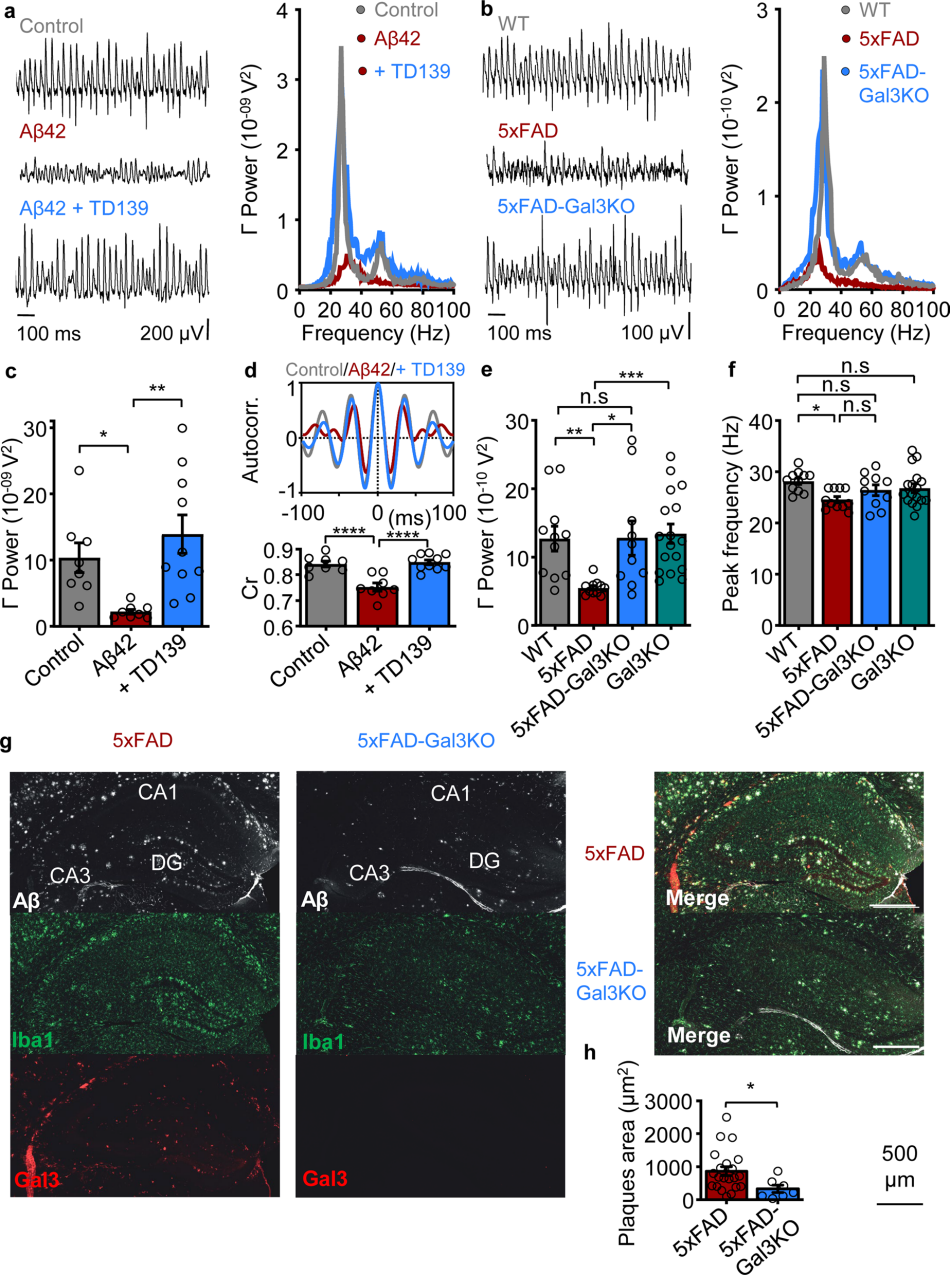
Involvement of gal3 in the disruption of gamma oscillations in two different AD-related mouse models. **a** Left: Representative example traces of gamma oscillations recorded in an interface-type recording chamber in control conditions (gray) and in slices pre-incubated for 15 min with 50 nM Aβ42 (red) or 50 nM Aβ42 + 10 µM TD139 (blue). Right: Representative power spectra from network activity (0–100 Hz) in CA3 hippocampus for each condition. **b** Left: Representative example traces of gamma oscillations recorded in CA3 of hippocampal slices from WT (gray), 5 × FAD (red) and 5 × FAD-Gal3KO (blue) mice in a submerged-type recording chamber. Right: Representative power spectra from network activity (0–100 Hz) in CA3 hippocampus for each animal group. **c** Summary of gamma oscillation power (20–80 Hz) showing that TD139 counteracted Aβ42-induced decrease of gamma power (control: 10.4 ± 2.24 × 10^–09^ V^2^, *n* = 8, *N* = 4; Aβ42: 2.23 ± 0.36 × 10^–09^ V^2^, *n* = 8, *N* = 4;* P* = 0.0478 vs control,* P* = 0.0040 vs Aβ42 + TD139, Aβ42 + TD139: 13.9 ± 2.9 × 10^–09^ V^2^, *n* = 10, *N* = 3;* P* = 0.2809 vs control). **d** Summary of the Cr showing that TD139 prevented Aβ42-induced deterioration of gamma rhythmicity (control: 0.84 ± 0.01, *n* = 8, *N* = 4; Aβ42: 0.75 ± 0.02, *n* = 8, *N* = 4; *P* < 0.001 vs control, *P* < 0.0001 vs Aβ42 + TD139, Aβ42 + TD139: 0.85 ± 0.01, *n* = 10, *N* = 3;* P* = 0.7034 vs control). Inset: Representative example of the autocorrelation function performed on gamma oscillations recorded in the conditions mentioned in **a**. **e** Quantification of gamma oscillation power (20–80 Hz) showing that the absence of gal3 signaling in 5 × FAD mice prevented the gamma power reduction typical of this AD mouse model and resulted in gamma power similar to WT mice (WT: 1.27 ± 0.18 × 10^–09^ V^2^, *n* = 11, *N* = 4, 5 × FAD: 0.55 ± 0.03 × 10^–09^ V^2^, *n* = 11, *N* = 4, 5 × FAD-Gal3KO: 1.28 ± 0.25 × 10^–09^ V^2^, *n* = 10, *N* = 4). Note that deletion of gal3 in isolation did not affect gamma oscillation power in the Gal3KO mice: 1.34 ± 0.14 × 10^–09^ V^2^, *n* = 17, *N* = 4. Statistics performed: Kruskal–Wallis test followed by Dunn’s multiple comparisons. A summary is provided in Additional file 2: Table S3. **f** Summary of peak frequency revealing that 5 × FAD mice had slower gamma oscillations (WT: 28.2 ± 0.61 Hz, *n* = 11, *N* = 4, 5 × FAD: 24.6 ± 0.58 Hz, *n* = 11, *N* = 4, 5 × FAD-Gal3KO: 26.4 ± 1.05 Hz, *n* = 10, *N* = 4, Gal3KO: 26.8 ± 0.87 Hz, *n* = 17, *N* = 4). Statistics performed: ordinary one-way ANOVA followed by Holm-Sidak’s multiple comparisons test. A summary is provided in Additional file 2: Table S4. In parallel to the lower power and slower central frequency of gamma oscillation, 5 × FAD mice displayed gamma oscillations with larger frequency variance (WT: 7.43 ± 0.48 Hz, *n* = 11, *N* = 4, 5 × FAD: 10.9 ± 0.69 Hz, *n* = 10, *N* = 4, 5 × FAD-Gal3KO: 7.55 ± 0.64 Hz, *n* = 10, *N* = 4, Gal3KO: 8.52 ± 0.62 Hz, *n* = 17, *N* = 4). Statistics performed: Kruskal–Wallis test followed by Dunn’s multiple comparisons. A summary is provided in Additional file 2: Table S5 and Additional file 1: Fig. S9. **g** Left panels, from top to bottom: Aβ (in grey) plaque size was reduced in CA3 area of the 5 × FAD-Gal3KO mouse hippocampus. Microglia (Iba1 in green) surrounding the plaques in both 5 × FAD and 5 × FAD-Gal3KO. Labelling corresponding to the specific antibody against Gal3 (in red) was abolished in 5 × FAD-Gal3KO mouse hippocampus. Right panels: merge of the labeling on the left panels. Scale bar 500 μm. DG: Dentate gyrus. **h** Quantification of the mean area of Aβ plaque labelling in CA3 area of 6-month-old mice in both 5 × FAD (864 ± 135.7 μm^2^, *n* = 21, *N* = 3) and 5 × FAD-Gal3KO (328.9 ± 112.7 μm^2^, *n* = 7, *N* = 3). *P* = 0.0225, two-tailed Mann Whitney test. *n*: number of plaques; *N*: number of mice per group. Data are presented as mean ± SE. Significance levels are shown as *P < 0.05, **P < 0.01, ***P < 0.001. n.s.: no significant statistical difference. In panels **c**–**f**, *n*: number of slices, *N*: number of animals

First, we performed recordings of gamma oscillations in the CA3 area of WT mouse hippocampal slices previously incubated with Aβ42 (“acute Aβ mouse model”) or Aβ42 + TD139. Recordings were performed in the same configuration as in the experiments in Fig. 1 (interface-type recording chamber, gamma oscillations induced after 15 min of incubation with 50 nM Aβ42 or 50 nM Aβ42 + 10 µM TD139 and recorded 30 min after rhythm induction). In slices pre-incubated with Aβ42, gamma oscillation power and rhythmicity were significantly decreased, in accordance with previous reports [51, 53, 62, 70, 71]. Surprisingly, co-incubation with TD139 fully prevented this Aβ42-induced functional impairment (Fig. 5a, c, d).

Second, we tested whether gamma oscillations are impaired in a familial AD mouse model also (“chronic 5×FAD mouse model”), similar to what we had demonstrated recently in another chronic AD model (App^NL−G−F^; [25]), and, if so, whether gal3 antagonism would then rescue network performance. 5×FAD mice exhibit a dramatically accelerated Aβ42 production with visible Aβ deposits starting as early as 2 months of age (Aβ plaques surrounded by gal3 + microglia) [27] and progressive cognitive deficits as early as 4–5 months of age [77, 78]. Notably, gal3 deficiency reduces Aβ plaque burden and plaque size, and improves cognitive performance in 5×FAD-Gal3KO mice at 6 months of age in hippocampal area CA1 and thalamus [27].

In the present study, we observed that the hippocampal CA3 network of 6-month-old 5×FAD mice exhibited a significant decrease of gamma oscillation power (Fig. 5e and Additional file 2: Table S3) and a shift of the peak gamma frequency toward lower values (Fig. 5f and Additional file 2: Table S4). This gamma slowing was accompanied by widening of the power spectra evidenced by the increase of the frequency variance (Additional file 1: Fig. S9, and Additional file 2: Table S5). Notably, in age-matched 5×FAD-Gal3KO mice, gamma oscillation power, peak frequency and frequency variance were unaffected and remained at the WT levels. Gal3 deletion did not cause further changes in these parameters (Fig. 5b, e, f, Additional file 1: Fig. S9 and Additional file 2: Tables S3–S5). Thus, the effects of gal3 paralleled those of Aβ42 on gamma oscillations as previously reported [51–53, 62, 70, 71]. This toxicity was prevented by gal3 inhibition (Fig. 1), and gal3 deletion prevented functional impairment of the gamma network rhythm in the 5×FAD mouse model of AD. Moreover, in the current study, we observed that gal3 deletion decreased the plaque size in hippocampal CA3 area of the 6-month-old 5×FAD mice (Fig. 5g, h).

Taken together, our results suggest that gal3 not only governs the microglial neuroinflammatory response as we previously demonstrated [27], but is also directly connected to AD-related aberrant synchronization in neuronal networks. Importantly, we can pharmacologically rescue this aberrant neuronal network behavior by intervening at the CRD site of gal3. This suggests gal3 as a potential therapeutic target effective not only against neurodegenerative processes, but also in rescuing normal neuronal signaling and cognition-relevant brain rhythms, thereby having a positive effect against cognitive decline.

## Discussion

Brain oscillations have been largely documented in humans aided by the use of EEG recordings [79–84] and are on the focus as a diagnostic tool. Their manipulations have emerged as a putative therapeutic approach for AD. Particularly, gamma oscillations hold a prominent interest in AD, due to studies performed both in vivo and ex vivo [73, 74, 82, 83, 85–89]. The mechanisms and gain-of-function found in these studies strengthen the notion that studying the neuronal network dynamics underlying gamma oscillations in different models provides useful tools as targets or effectors that may have therapeutic potential. Moreover, these studies reveal that neuronal network dynamics in animal studies and in humans may operate in a way similar to that observed experimentally ex vivo. For instance, the application of acetylcholine to brain slices increases neural excitability, mimicking cholinergic inputs to the hippocampus during exploratory behaviour in vivo [90]. In addition, cholinergic gamma oscillations recorded ex vivo share numerous mechanistic commonalities with in vivo gamma oscillations, involving glutamatergic-mediated excitation and fast rhythmic GABAergic inhibition [59, 90, 91] similar to kainate-induced gamma oscillations ex vivo [25, 51–53, 55, 61, 92].

Thus, synchronized fast neuronal activity between PCs and FSNs within the hippocampal CA3 circuits gives rise to the emergence of periodic fluctuations of the LFP in the gamma frequency band. Such activities comprise phase-locked AP firing and rhythmic inhibitory and excitatory postsynaptic currents (IPSCs and EPSCs, respectively), as shown in the current study. Particularly, FSNs command the entrainment of PC activity during gamma oscillations and provide stability of the neuronal network performance. Therefore, FSNs are suitable targets to counteract deficient cognitive performance [8, 9, 16, 25, 54, 55, 68, 75, 93]. The coordinated and cooperative activity within the network allows for the binding of neurons in neuronal ensembles that then process sensory information, thus supporting cognitive processes and memory formation [94]. Therefore, any functional deviation such as impairment of FSN activity, disruption of inhibitory and excitatory synaptic transmission, and consequent aberrant PC activity, may negatively impact the overall network performance, with detrimental cognitive implications as observed in AD.

In this regard, we have previously observed that Aβ42 induces synaptic failure as well as desynchronization of AP firing of PCs [51, 53], degradation of FSN spike phase-locking [52, 62] relative to gamma oscillations, and overall neuronal network impairment [51, 52, 62, 70, 71, 95]. Interestingly, the current study revealed for the first time that acute gal3 induces a functional network collapse that shares commonalities with the reported effects of Aβ42 on neuronal networks dynamics [51–53, 62, 89]. Moreover, a possible association between gal3 and Aβ42 underlying detrimental microglial activation in AD has been described recently [27]. Despite huge efforts towards counteracting the pathological events leading to progressive cognitive impairment in AD, disease-modifying approaches have failed or shown only modest advances [76]. One possible shortcoming of past therapeutic attempts is the fact that their focus has been merely on Aβ42 plaque removal [76], whereas growing evidence points to neuronal synchronization as well as neuroinflammation as critical targets to counteract the progression of AD [26–29]. Circuit entrainment into physiological brain rhythms has shown prominence as a suitable and promising approach counteracting progressive neuronal network dysfunction and deficient cognitive performance in AD, with a focus on FSN activity [52, 73, 75]. On the other hand, microglial gal3 has been proposed as a central regulator of microglia-driven neuroinflammatory responses in AD [27]. We found here that inhibition or deletion of gal3 counteracts all of the functional deviations it induces (e.g., decrease of gamma power and rhythmicity, increase of gamma frequency variance, impairment of PC and FSN firing rate and phase-lock to gamma oscillation, hyperpolarization of FSN resting membrane potential, increase of the firing threshold in basal conditions, and disruption of rhythmic inhibitory and excitatory synaptic transmission), thus preserving normal neuronal network dynamics that are relevant for cognitive capabilities.

This functional impact of gal3 on neighboring neurons and synapses farther from Aβ42 plaques should also be considered. Here, we observed that preincubation of the hippocampal neuronal network with gal3 induced a drastic degradation of gamma oscillations mediated by gal3 CRD. Interestingly, gal3 has been reported to bind to and activate different microglial receptors, which include TREM2 [27] and TLR4 [33]. Particularly, gal3 binding to TREM2 and TLR4 has been described to be mediated by its CRD [27, 33]. Accordingly, the consistent prevention of gal3-mediated disruption of neuronal and network function observed in the presence of the gal3 inhibitor TD139 reinforces our findings since TD139 is a 3,3’-Bis-(4-aryltriazol-1-yl) thio-digalactoside gal3 inhibitor with high affinity for gal3 CRD.

In this study, we tested whether the disruption of gamma oscillations observed following exposure of the hippocampal network to gal3 is accompanied by changes in the expression of some genes related to microglial activity. Overall, we did not observe relevant changes in the expression of the analyzed genes, which could be ascribed to the exposure time (45 min total), which appears long enough to prevent an efficient induction of gamma oscillations but too short to trigger a significant change of gene expression. However, the overall quantification of the expression of the listed transcripts in the present study should just be taken as a descriptive clue and future experiments should be performed to further assess a wider range of microglia activation-related genes with longer exposure to gal3.

The electrophysiological changes observed here in neurons and the network appear to rely on a mechanism linked to the cell membrane (i.e., a particular microglial receptor), which probably induces an early microglial activation with further consequences if the application lasts longer, as expected from the time-dependence shown in Fig. 1 versus Fig. 2, and as it may happen in vivo during sustained gal3 release. Again, the overall preventive effect of TD139 reinforces this notion. TD139 mainly acts extracellularly within the time of application employed here. The inhibitor does not reach intracellular compartments unless incubation/wash-in lasts 24 h or longer [96].

Furthermore, we found that gal3 is less efficient in disrupting gamma oscillations if the network has already properly entrained into the gamma rhythm (see Fig. 2). However, applied to the network prior to FSN engagement into strong spike-phase coupling, gal3 prevented the proper establishment of a coordinated activity that led to gamma oscillations of physiological relevance (see Fig. 4). In an AD scenario, it is tempting to hypothesize that at a certain point during very early pathology progression, cognition-relevant neuronal networks preserve their functionality due to homeostatic mechanisms (a high concentration of gal3 is needed to disrupt cellular and neuronal normal activities). As the pathology progresses, such homeostatic mechanisms become overwhelmed, perhaps even deleterious (i.e., microglial shift to damage-associated microglia phenotype) [22] and gal3 concentration largely increases. Then, a progressively weakened neuronal network appears more susceptible to gal3, which could also reach non-damaged neurons/synapses by diffusing throughout the brain parenchyma, once secreted by the already activated microglia. At this point, a lesser gal3 concentration could drive major dysfunction, which is in line with the damage amplification role of gal3. Such hypothesis deserves further experimental confirmations.

A plausible explanation for the broad range of gal3-induced effects observed could be found in the complex purinergic signaling in the neuron-microglia crosstalk. It has been proposed that the initial increase of extracellular adenosine to levels far greater than reached in physiological conditions initially leads to a burst of adenosine receptor 1 (A_1_R)-mediated inhibition, and the continuous massive overflow of extracellular adenosine then overcomes the restricted activation of A_2A_Rs, which results in a predominant role of A_2A_Rs in the development of neurodegeneration [97]. In line with this proposal, a paramount impact of ATP/adenosine signaling on hippocampal circuitry function has been observed. At mossy fiber-CA3 synapses, microglia-derived ATP differentially modulates synaptic transmission and short-term plasticity through activation of presynaptic P2X4 receptors and A_1_R, respectively, with the latter converting to adenosine extracellularly [98]. Additionally, blockade of A_2A_Rs prevents lipopolysaccharide-induced impairment of long-term potentiation in rats in vivo, by counteracting the shift of microglia towards a pro-inflammatory phenotype [99]. Notably, A_2A_R is upregulated in the APP/PS1 mouse model [100] as well as in cortical areas [101] and the hippocampal formation of AD patients [102]. It has been found that adenosine inhibits KA-induced and spontaneous gamma oscillations, particularly via the activation of A_1_R [103]. Consistent with our hypothesis and results, it has been observed that endogenous ATP release drastically reduces PC AP firing rate as well as spike synchronization during KA-induced gamma oscillations in the CA3 area. Moreover, both KA- and acetylcholine-induced gamma oscillations are inhibited by ATP/adenosine receptor activation [104]. Here we also observed that gal3 impairs cholinergic-induced gamma oscillations (Additional file 1: Fig. S1), which indicates that the effect of gal3 is independent of the method of gamma oscillation induction. Of note, Aβ42 also induces degradation of cholinergic gamma oscillations and gamma-theta rhythm interaction in the hippocampus [62]. In addition, the degeneration of basal forebrain cholinergic neurons (one of the relevant cholinergic inputs to and for the hippocampal rhythmogenesis) has been ascribed as a common feature of AD [105].

In our study, we found impairment of both excitatory and inhibitory synaptic drive onto PCs as well as a decrease of excitatory input onto FSNs. Interestingly, gal3 differentially affected the amplitude or frequency of excitatory synaptic events in FSNs compared to PCs, which suggested that gal3 affects synaptic transmission in a synapse-specific manner with differential effects and sites of action (presynaptic and postsynaptic sites), depending on the synapse type and the circuit activity. In this regard, hippocampal A_1_Rs are known to hyperpolarize FSNs, reduce the excitability of PCs and interneurons, and also reduce neurotransmitter release [106, 107]. Changes in neurotransmitter release are mostly associated with changes of frequency of synaptic events, observed in this study as a decrease of EPSCs onto PCs (Fig. 3b) and FSNs in either basal or activated state once the circuit is challenged with gal3 prior to rhythm entrainment (Additional file 1: Fig. S6e and 7b). Notably, gal3 reduced the occurrence of larger IPSC components in PCs probably as a reflection of the observed FSN impairment since the major perisomatic inhibition of PCs is driven by FSNs [8, 9]. The overall collapse of the network was also observed in the gal3-induced increased variability of the phase relation of both EPSCs and IPSCs with the corresponding LFP-gamma as well as the loss of rhythmicity of the postsynaptic currents. This loss of rhythmicity of synaptic transmission likely accounts for the degradation of the network rhythm since the generation of gamma oscillations depends on balanced excitatory and inhibitory interplay [12]. However, due to the diverse evidence of purinergic signaling in the modulation of the operational capacity of the hippocampal circuitry, and commonalities found in our study regarding gal3-induced neuronal network collapse, a possible underlying mechanism involving ATP/adenosine receptor activation deserves further research, without exclusion of an indirect involvement of astrocytes, nitric oxide, metabolic arrestment [31, 32, 102, 108] and a direct effect of gal3 on PCs and FSN. Also, effects of microglial activation due to the slicing procedure appear negligible in our study since microglia seem to be less relevant for moderate tissue repair at the slice cut surfaces as well as for synaptic remodelling and neuronal network formation, at least during the second and third postnatal weeks of hippocampal maturation in situ [109, 110]. Moreover, application of the gal3 specific inhibitor TD139 alone (Fig. 1c, d), or deletion of gal3 (Fig. 5e, f), did not affect neuronal network dynamics prior to gamma oscillation induction.

Finally, there is mounting evidence suggesting that in AD, synaptic and network failure starts long before the establishment of Aβ42 deposits into solid plaques and manifestation of cognitive decline [22, 74, 111–113]. Interestingly, in the APP/PS1 mouse model, associative long-term synaptic plasticity is impaired in CA3 PCs at the early onset of AD-like features due to the postsynaptic activation of upregulated A_2A_R [100]. Moreover, focal glial activation has been reported to precede amyloid plaque deposition in APP transgenic mice associated with a vicious cycle of APP proteolytic cleavage that gives rise to soluble and amyloidogenic immunostimulatory mediators [28]. Using a proteomic approach, it has been found that immune alterations in microglia in 5×FAD mice occur prior to plaque deposition [114].

Recently, direct involvement of microglia-released gal3 and Aβ cross-seeding agents in plaque formation has been validated [27, 115, 116]. Particularly, the 5×FAD mouse model lacking gal3 (5×FAD-Gal3KO) fails to develop prominent Aβ plaques and cognitive impairment typical of the 5×FAD model at 6 months of age [27]. Here we found that a possible underlying functional reason explaining these previous reports could be the degradation of gamma oscillations (observed in the 5×FAD model at 6 months of age), which is absent in the age-matched 5×FAD-Gal3KO mice that also show significant reduction of Aβ load in the hippocampal CA3 area (Fig. 5). 5×FAD mice also showed a significant slowing of gamma oscillation frequency compared to WT control, but the age-matched 5×FAD-Gal3KO retained values similar to WT. Interestingly, slower gamma oscillations have been observed also in the CA3 area of organotypic hippocampal cultures under microglial priming with interferon gamma [117]. Inhibition of gal3 by co-application of TD139 prevented the decreases of both gamma oscillation power and frequency in the acute Aβ application model (see Fig. 5). 5×FAD mice also displayed gamma oscillations of higher frequency variance (Additional file 1: Fig. S9), which is in line with the less rhythmic behavior observed during gal3 acute application (e.g., see Fig. 1d, f). This decrease of gamma rhythm quality was absent in 5×FAD mice lacking gal3. Interestingly, acute Aβ decreases gamma rhythm quality evidenced by a decrease of the Cr (Fig. 5c) [51, 55] or an increase of the frequency variance observed under acute Aβ application [52] or in the App^NL−G−F^ mouse model [25]. This suggests a highly congruent parallelism between the underlying mechanisms of gal3- and Aβ-induced degradation of gamma oscillations. Moreover, we have observed that the cognitive impairment correlates with the impairment of gamma oscillations ex vivo [25]. Recently, we found that the recovery of cognitive functions is accompanied by recovery of the hippocampal oscillatory activity and that counteracting proinflammatory triggers counteracts degradation of gamma oscillations in our model [61, 92].

### Conclusion

In summary, here we report for the first time that gal3, a proposed central microglial/neuroinflammatory regulator in AD, causes degradation of a neuronal network rhythm and reveal its underlying neuronal synchronization mechanisms by performing *ex-vivo* recordings of gamma oscillations, which may well serve as an appropriate prototype for cognition-relevant neuronal network dynamics. The impairments observed are mediated by the gal3-CRD and prevented by the gal3 inhibitor TD139 in a dose-dependent manner. Additionally, gal3 prevents neuronal network entrainment into proper gamma rhythm. Such disruption is accompanied by the impairment of FSN and PC gamma spike-phase locking and disturbances of excitatory and inhibitory synaptic transmission. Interestingly, we found a possible functional link for gal3 to AD since (1) TD139 prevents Aβ42-induced degradation of gamma oscillations ex vivo and (2) gamma oscillations are impaired in the hippocampal CA3 area of the 5×FAD mouse model at 6 months of age while gamma oscillations recorded from 5×FAD mice lacking gal3 (5×FAD-Gal3KO) remain similar to age-matched WT counterparts. In parallel, 5×FAD-Gal3KO display lower Aβ plaques in the recorded hippocampal CA3 area. Moreover, our results bridge the gap between cellular and molecular notions on the central role of gal3 in AD progression and behavioral studies by providing functional evidence that is relevant for those behaviors. This reinforces the therapeutic potential of inhibiting/removing gal3 to counteract AD progression and putatively as disease-modifying interventions for other neurodegenerative disorders involving microglial activation and neuroinflammation. In vivo recordings performed at different time points during disease progression (e.g., in the App^NL−G−F^ mouse model [25] lacking gal3 signaling) will ultimately cross-validate the therapeutic potential of our current findings. Moreover, such studies on the follow-up of the molecular and cognitive changes linked to the functional parameters studied here, will establish a timely rescue approach including effective inhibitor dosage, safety, and treatment schedule, and will further our notions on the therapeutic potential of gal3 inhibition in AD.

## Supplementary Information

**Additional file 1: Fig. S1** Electrophysiological distinction of pyramidal cells and fast-spiking interneurons. **Fig. S2** Gal3 induces decrease of gamma oscillation power of cholinergic-induced gamma oscillations. **Fig. S3** Effects of 1 µM gal3 on FSN-gamma phase-lock from the concomitant recordings performed in Fig. 2a. **Fig. S4.** Effects of 2 µM gal3 on FSN EPSC rhythmicity and gamma-EPSC relationship. **Fig. S5** Effect of 1 µM gal3 on FSN firing threshold and spontaneous AP firing in basal state. **Fig. S6** Effect of 1 µM gal3 on excitatory input to FSN in basal state. **Fig. S7** Excitatory input onto FSN in activated state 45 min after gal3 application. **Fig. S8** Gene expression levels in brain slices treated with gal3 prior to gamma oscillation induction. **Fig. S9** Frequency variance (calculated from recordings of gamma oscillations in Fig. 5e and f) is increased in 6 months-old 5 × FAD mice.

**Additional file 2: Table S1.** Summary of statistics performed in Fig. 1c. **Table S2.** Summary of statistics performed in Fig. 1d. **Table S3.** Summary of statistics performed in Fig. 5e. **Table S4.** Summary of statistics performed in Fig. 5f. **Table S5.** Summary of statistics performed in Fig. 5e and f for frequency variance shown in Additional file 1: Fig. S9.

## Data Availability

All the data are provided in the figure legends, in the supplementary material and available upon request without restrictions.

## Abbreviations

AP: Action potentials
Aβ42: Beta-amyloid peptide
Cr: Coefficient of rhythmicity
CRD: Carbohydrate recognition domain
DG: Dentate gyrus
EPSC: Excitatory postsynaptic current
FSN: Fast-spiking interneuron
gal3: Galectin 3
IPSC: Inhibitory postsynaptic current
KA: Kainic acid
LFP: Local field potential
PC: Pyramidal cell
TLR4: Toll-like receptor 4
TREM2: Triggering receptor expressed on myeloid cells 2
XC: Cross-correlation index
