# Supplementary material for: Targeting galectin-3 to counteract spike-phase uncoupling of fast-spiking interneurons to gamma oscillations in Alzheimer’s disease
Source: Transl Neurodegener. 2023 Feb 6;12:6. doi: 10.1186/s40035-023-00338-0 (PMC9901156; doi:10.1186/s40035-023-00338-0)
Supplement: Supplementary file 1 — Additional file 1: Fig. S1 Electrophysiological distinction of pyramidal cells and fast-spiking interneurons. Fig. S2 Gal3 induces decrease of gamma oscillation power of cholinergic-induced gamma oscillations. Fig. S3 Effects of 1 µM gal3 on FSN-gamma phase-lock from the concomitant recordings performed in Fig. 2a. Fig. S4. Effects of 2 µM gal3 on FSN EPSC rhythmicity and gamma-EPSC relationship. Fig. S5 Effect of 1 µM gal3 on FSN firing threshold and spontaneous AP firing in basal state. Fig. S6 Effect of 1 µM gal3 on excitatory input to FSN in basal state. Fig. S7 Excitatory input onto FSN in activated state 45 min after gal3 application. Fig. S8 Gene expression levels in brain slices treated with gal3 prior to gamma oscillation induction. Fig. S9 Frequency variance (calculated from recordings of gamma oscillations in Fig. 5e and f) is increased in 6 months-old 5 × FAD mice. [file 40035_2023_338_MOESM1_ESM.docx]

***Additional File 1.***

**SUPPLEMENTARY FIGURES**

**Supplementary Figure S1** Electrophysiological distinction of pyramidal cells and fast-spiking interneurons. **a** Representative somatic shape and characteristic electrical responses to different stimulation protocols for Glutamatergic pyramidal cells recorded in the *str. pyramidale* of area CA3 of the hippocampus or from *str. radiatum* for fast-spiking GABAergic inhibitory interneurons **(b)**. **i)** 2 steps protocol **ii)** elicit burst protocol, **iii**) ramp protocol, and the electrical response in the different populations analyzed.

**Supplementary Figure S2** Gal3 induces decrease of gamma oscillation power of cholinergic-induced gamma oscillations. Gamma oscillations were induced by bath applying 10 μM Ach + 2 μM Phys (*see methods*). Recordings were performed in an interface-type recording chamber in control conditions (no gal3) and from slices incubated 15 min with 1 μM gal3. Similar to the KA-induced gamma oscillations, prior to the recordings, gamma oscillations were allowed to stabilize for at least 30 min. **a** Summary of gamma oscillations power in control conditions (gray, 2.34 ± 0.6 x 10^-09^ V^2^, n= 7, N= 3) and after 15 min incubation with gal3 (red, 0.9 ± 0.12 x 10^-09^ V^2^, n= 8, N= 3; p= 0.0289). **b** **Summary** of gamma oscillations peak frequency in the conditions described in A (control: 37.3 ± 1.72 Hz, n= 7, N= 3; gal3: 34.5 ± 1.66 Hz, n= 8, N= 3; p= 0.4435). **c** Summary of frequency variance calculated from gamma oscillations in the conditions described in A (control: 12.1 ± 3.56 Hz, n= 7, N= 3; gal3: 17.9 ± 3.66 Hz, n= 8, N= 3; p= 0.2810). Statistics performed: two-tailed Mann Whitney test. Data are presented as a mean ± SE. Significance level is shown as * p<0.05. n. s: no significant statistical difference, n: number of slices, and N: number of animals.

**Supplementary Figure S3** Effects of 1µM gal3 on FSN-gamma phase-lock from the concomitant recordings performed in Fig. 2a. **a** Summary of resultant vector length in control conditions and 30 min after gal3 application (control: 0.56 ± 0.08, gal3: 0.43 ± 0.1, n= 6, N= 3; p= 0.2188). **b** Quantification of the preferred gamma phase-angle (control: 5.33 ± 0.15 radians, gal3: 4.63 ± 0.73 radians, n= 6, N= 3; p>0.9999). **c** Effect of gal3 on FSN firing rate (control: 5.59 ± 1.32 Hz, gal3: 6.54 ± 1.96, n= 6, N= 3; p= 0.8438). Statistics performed: two-tailed Wilcoxon test. Data are presented as a mean ± SE. n. s: no significant statistical differences, n: number of cells, N: number of animals.

**Supplementary Figure S4** Effects of 2µM gal3 on FSN EPSC rhythmicity and gamma-EPSC relationship. **a** Summary of Cr as a measure of EPSC quality within the gamma frequency (control: 0.82 ± 0.01, gal3: 0.78 ± 0.02, n= 7, N= 4; p= 0.0156). **b** Quantification of the peak from EPSC-LFP cross-correlation analysis evidencing gal3-induced impairment of FSN EPSC and gamma rhythm similarities (control: 0.44 ± 0.09, gal3: 0.27 ± 0.09, n= 7, N= 4; p= 0.0156). **c** Summary of the cross-correlation peak lag (control: -4.74 ± 0.75 ms, gal3: -3.37 ± 4.71 ms, n= 7, N= 4; p= 0.5781). Statistics performed: two-tailed Wilcoxon test. Data are presented as a mean ± SE. Significance level is shown as * p<0.05. n. s: no significant statistical differences, n: number of cells, N: number of animals.

**Supplementary Figure S5** Effect of 1µM gal3 on FSN firing threshold and spontaneous AP firing in basal state. **a-c** show summary bar graphs of firing threshold in control conditions monitored over 15 min (basal: -43.6 ± 0.82 mV, ACSF: -44.2 ± 1.05 mV, n= 17, N= 6; p= 0.4386), after gal3 (basal: -45.4 ± 2.14 mV, gal3: -40.2 ± 1.25 mV, n= 10, N= 7; p= 0.0113) or gal3 + TD139 (basal: -44.3 ± 1.84 mV, gal3 + TD139: -42.4 ± 1.12 mV, n= 10, N= 5; p= 0.1516), respectively. Statistics performed: two-tailed paired t-test. **d-f** show the firing rate of FSN that were firing APs in basal state in control conditions monitored over 15 min (basal: 0.21 ± 0.07 Hz, ACSF: 0.32 ± 0.09 Hz, n= 12, N= 5; p= 0.1625, two-tailed paired t-test), after gal3 (basal: 0.58 ± 0.19 Hz, gal3: 0.37 ± 0.14 Hz, n= 7, N= 5; p= 0.0156, two-tailed Wilcoxon test) and gal3 + TD139 (basal: 0.69 ± 0.31 Hz, gal3 + TD139: 0.61 ± 0.17 Hz, n= 5, N= 3; p>0.9999, two-tailed Wilcoxon test), respectively. Data are presented as a mean ± SE. Significance level is shown as * p<0.05. n. s: no significant statistical differences, n: number of cells, N: number of animals.

**Supplementary Figure S6** Effect of 1µM gal3 on excitatory input to FSN in basal state. **a** Summary of EPSC amplitude in control conditions (basal: 9.75 ± 1.28 pA, ACSF: 9.0 ± 1.38 pA, n= 17, N= 6; p= 0.3841). **b** Summary of EPSC frequency in control conditions (basal: 29.6 ± 1.1 Hz, ACSF: 29.9 ± 1.15 Hz, n= 17, N= 6; p= 0.5293). **c** Quantification of EPSC charge transfer in control conditions (basal: 115.8 ± 17.5 pC, ACSF: 108.6 ± 17.9 pC, n= 17, N= 6; p= 0.5372). **d** Summary of gal3 effect on EPSC amplitude (basal: 11.9 ± 1.72 pA, gal3: 11.2 ± 1.73 pA, n= 10, N= 7; p= 0.4209). **e** Summary of gal3 effect on EPSC frequency (basal: 34.1 ± 0.79 Hz, gal3: 32.7 ± 0.97 Hz, n= 10, N= 7; p= 0.0204). **f** Quantification of gal3 effect on EPSC charge transfer (basal: 162.7 ± 26.9 pC, gal3: 149.6 ± 27.4 pC, n= 10, N= 7; p= 0.2567). **g** Summary of gal3 + TD139 effect on EPSC amplitude (basal: 9.02 ± 1.73 pA, gal3: 8.32 ± 1.41 pA, n= 10, N= 5; p= 0.4687). **h** Summary of gal3 + TD139 effect on EPSC frequency (basal: 31.4 ± 1.79 Hz, gal3 + TD139: 30.6 ± 1.66 Hz, n= 10, N= 5; p= 0.2689). **i** Quantification of gal3 + TD139 effect on EPSC charge transfer (basal: 106.7 ± 24.3 pC, gal3 + TD139: 100.8 ± 21.9 pC, n= 10, N= 5; p= 0.5679). Statistics performed: two-tailed paired t test. Data are presented as a mean ± SE. Significance level is shown as * p<0.05. n. s: no significant statistical differences. n: number of cells, N: number of animals. Insets show representative example traces for each condition.

**Supplementary Figure S7** Excitatory input onto FSN in activated state 45 min after gal3 application. **a** Summary of gal3 effect on EPSC amplitude (ACSF: 17.5 ± 2.39 pA, n= 12, N= 6; gal3: 22.1 ± 3.47 pA, n= 13, N= 7; p>0.9999 vs. control, p> 0.9999 vs. gal3 + TD139, gal3 + TD139: 23.0 ± 5.03 pA, n= 10, N= 5; p> 0.9999 vs. control, Kruskal-Wallis test followed by Dunn’s multiple comparisons test). **b** Summary of gal3 effect on EPSC frequency (ACSF: 36.5 ± 0.73 Hz, n= 12, N= 6; gal3: 32.4 ± 0.95 Hz, n= 14, N= 7; p= 0.0040 vs. control, p= 0.0018 vs. gal3 + TD139, gal3 + TD139: 23.0 ± 5.03 Hz, n= 10, N= 5; p= 0.5571 vs. control, ordinary one-way ANOVA followed by Holm-Sidak’s multiple comparisons test). **c** Quantification of charge transfer for each condition (ACSF: 253.6 ± 34.9 pC, n= 12, N= 6; gal3: 327.9 ± 62.6 pC, n= 14, N= 7; p> 0.9999 vs. control, p> 0.9999 vs. gal3 + TD139, gal3 + TD139: 349.5 ± 81.6 pC, n= 10, N= 5; p> 0.9999 vs. control, Kruskal-Wallis test followed by Dunn’s multiple comparisons test). Insets show representative example traces for each condition. **d** Quantification of EPSCs Cr showing that inhibition of gal3 signaling prevents gal3-mediated impairment of EPSC rhythmicity measured as Cr (ACSF: 0.82 ± 0.01, n= 11, N= 6; gal3: 0.69 ± 0.02, n= 14, N= 7; p< 0.0001 vs. control, p< 0.0001 vs. gal3 + TD139, gal3 + TD139: 0.84 ± 0.01, n= 10, N= 5; p= 0.4115 vs. control). **e** Summary of the maximal correlation between FSN EPSCs and concomitant gamma oscillations (ACSF: 0.48 ± 0.07, n= 12, N= 6; gal3: 0.44 ± 0.08, n= 14, N= 7; p= 0.7539 vs. control, p= 0.6088 vs. gal3 + TD139, gal3 + TD139: 0.56 ± 0.08, n= 10, N= 5; p= 0.7539 vs. control). **f** Phase relationship between FSN EPSCs and concomitant gamma oscillations (ACSF: -4.18 ± 0.44 ms, n= 12, N= 6; gal3: -1.33 ± 2.45 ms, n= 14, N= 7; p= 0.5387 vs. control, p= 0.6376 vs. gal3 + TD139, gal3 + TD139: -3.42 ± 0.56 ms, n= 10, N= 5; p= 0.7644 vs. control). Statistics performed in D-F: ordinary one-way ANOVA followed by Holm-Sidak’s multiple comparisons test. Data are presented as a mean ± SE. Significance levels are shown as ** p<0.01, **** p< 0.0001. n. s: no significant statistical differences. n: number of cells, N: number of animals.





**Supplementary Figure S8** Gene expression levels in brain slices treated with gal3 prior to gamma oscillation induction. Expression levels of the mRNAs of **a)** *Trem2*, **b)** *Tlr4*, **c)** *Clec7a*, **d)** *Cx3cr1* and **e)** *Gfap* in frozen hippocampal slices from WT mice under the different conditions taken at the end of recordings performed in Fig. 4: Control (ACSF), Control KA, 1 µM Gal3 and 1 µM Gal3 + 10 µM TD139. No differences were observed in the levels of **a)** *Trem2* (ACSF: 7.58 ± 0.23, N= 3; Control KA: 7.14 ± 0.43, N= 5; gal3: 7.20 ± 0.43, N= 5; gal3 + TD139: 6.85 ± 0.58, N= 3; p= 0.8863), **b)** *Tlr4* (ACSF: 17.52 ± 0.58, N= 2; Control KA: 15.27 ± 0.82, N= 5; gal3: 15.76 ± 0.65, N= 4; gal3 + TD139: 15.81 ± 0.52, N= 3; p= 0.4455), and **c)** *Clec7a* (ACSF: 15.49 ± 0.40, N= 3; Control KA: 14.14 ± 0.42, N= 5; gal3: 14.80 ± 0.28, N= 5; gal3 + TD139: 13.73 ± 1.03, N= 3; p= 0.3982). However, statistical differences were found in the ΔCt levels of **d)** *Cx3cr1* (ACSF: 9.56 ± 0.44, N= 3; Control KA: 16.08 ± 1.11, N= 5; gal3: 8.37 ± 0.33, N= 4; gal3 + TD139: 7.58 ± 0.85, N= 3; p= 0.0004; Control KA vs gal3, p=0.046; Control KA vs gal3 + TD139, p= 0.0185) and **e)** *Gfap* (ACSF: 16.02 ± 0.63, N= 3; Control KA: 13.71 ± 1.17, N= 5; gal3: 12.55 ± 0.79, N= 5; gal3 + TD139: 10.86 ± 0.89, N= 3; p= 0.0344; Control vs gal3 + TD139, p= 0.0479). Data are presented as mean ± SE of two independent experiments, normalized to *Gapdh* and expressed as ΔCt. Kruskal-Wallis non-parametric test followed by Dunn’s multiple comparisons test was performed. Data are presented as a mean ± SE. Significance level is shown as * p<0.05. N: number of animals.

**Supplementary Figure S9.** Frequency variance (calculated from recordings of gamma oscillations in **Fig. 5e and f**) **is increased in 6 months-old 5xFAD mice.** Data is presented as a mean ± SE. Statistics performed: Kruskal-Wallis test followed by Dunn’s multiple comparisons test (see Additional file 1, Table S5). Significance levels are shown as ** p<0.01.
