# Supplementary material for: Targeting galectin-3 to counteract spike-phase uncoupling of fast-spiking interneurons to gamma oscillations in Alzheimer’s disease
Source: Transl Neurodegener. 2023 Feb 6;12:6. doi: 10.1186/s40035-023-00338-0 (PMC9901156; doi:10.1186/s40035-023-00338-0)
Supplement: Supplementary file 2 — Additional file 2: Table S1. Summary of statistics performed in Fig. 1c. Table S2. Summary of statistics performed in Fig. 1d. Table S3. Summary of statistics performed in Fig. 5e. Table S4. Summary of statistics performed in Fig. 5f. Table S5. Summary of statistics performed in Fig. 5e and f for frequency variance shown in Additional file 1: Fig. S9. [file 40035_2023_338_MOESM2_ESM.docx]

***Additional File 2.***

**SUPPLEMENTARY TABLES**

**Supplementary Table 1. Summary of statistics performed in Fig. 1C.**

| Number of families | 1 |  |  |  |  |  |  |  |
| --- | --- | --- | --- | --- | --- | --- | --- | --- |
| Number of comparisons per family | 15 |  |  |  |  |  |  |  |
| Alpha | 0.05 |  |  |  |  |  |  |  |
| **Holm-Sidak's multiple comparisons test** | **Mean Diff.** | **Significant?** | **Summary** | **Adjusted P Value** |  |  |  |  |
|  |  |  |  |  |  |  |  |  |
| Control KA vs. Gal3 | 1.037e-008 | Yes | *** | 0.0004 |  |  |  |  |
| Control KA vs. Gal3 + 10 uM TD139 | -3.772e-010 | No | ns | 0.9931 |  |  |  |  |
| Control KA vs. Gal3 + 3 uM TD139 | 8.831e-009 | Yes | * | 0.0103 |  |  |  |  |
| Control KA vs. Gal3 + 1 uM TD139 | 1.009e-008 | Yes | ** | 0.0014 |  |  |  |  |
| Control KA vs. 10 uM TD139 | -1.561e-009 | No | ns | 0.9931 |  |  |  |  |
| Gal3 vs. Gal3 + 10 uM TD139 | -1.075e-008 | Yes | *** | 0.0002 |  |  |  |  |
| Gal3 vs. Gal3 + 3 uM TD139 | -1.538e-009 | No | ns | 0.9931 |  |  |  |  |
| Gal3 vs. Gal3 + 1 uM TD139 | -2.788e-010 | No | ns | 0.9931 |  |  |  |  |
| Gal3 vs. 10 uM TD139 | -1.193e-008 | Yes | ** | 0.0014 |  |  |  |  |
| Gal3 + 10 uM TD139 vs. Gal3 + 3 uM TD139 | 9.208e-009 | Yes | ** | 0.0074 |  |  |  |  |
| Gal3 + 10 uM TD139 vs. Gal3 + 1 uM TD139 | 1.047e-008 | Yes | *** | 0.0009 |  |  |  |  |
| Gal3 + 10 uM TD139 vs. 10 uM TD139 | -1.184e-009 | No | ns | 0.9931 |  |  |  |  |
| Gal3 + 3 uM TD139 vs. Gal3 + 1 uM TD139 | 1.259e-009 | No | ns | 0.9931 |  |  |  |  |
| Gal3 + 3 uM TD139 vs. 10 uM TD139 | -1.039e-008 | Yes | * | 0.0127 |  |  |  |  |
| Gal3 + 1 uM TD139 vs. 10 uM TD139 | -1.165e-008 | Yes | ** | 0.0031 |  |  |  |  |
| **Test details** | **Mean 1** | **Mean 2** | **Mean Diff.** | **SE of diff.** | **n1** | **n2** | **t** | **DF** |
| Control KA vs. Gal3 | 1.196e-008 | 1.596e-009 | 1.037e-008 | 2.270e-009 | 14 | 13 | 4.567 | 59 |
| Control KA vs. Gal3 + 10 uM TD139 | 1.196e-008 | 1.234e-008 | -3.772e-010 | 2.228e-009 | 14 | 14 | 0.1693 | 59 |
| Control KA vs. Gal3 + 3 uM TD139 | 1.196e-008 | 3.134e-009 | 8.831e-009 | 2.612e-009 | 14 | 8 | 3.380 | 59 |
| Control KA vs. Gal3 + 1 uM TD139 | 1.196e-008 | 1.875e-009 | 1.009e-008 | 2.441e-009 | 14 | 10 | 4.134 | 59 |
| Control KA vs. 10 uM TD139 | 1.196e-008 | 1.353e-008 | -1.561e-009 | 2.876e-009 | 14 | 6 | 0.5426 | 59 |
| Gal3 vs. Gal3 + 10 uM TD139 | 1.596e-009 | 1.234e-008 | -1.075e-008 | 2.270e-009 | 13 | 14 | 4.733 | 59 |
| Gal3 vs. Gal3 + 3 uM TD139 | 1.596e-009 | 3.134e-009 | -1.538e-009 | 2.649e-009 | 13 | 8 | 0.5805 | 59 |
| Gal3 vs. Gal3 + 1 uM TD139 | 1.596e-009 | 1.875e-009 | -2.788e-010 | 2.479e-009 | 13 | 10 | 0.1124 | 59 |
| Gal3 vs. 10 uM TD139 | 1.596e-009 | 1.353e-008 | -1.193e-008 | 2.909e-009 | 13 | 6 | 4.100 | 59 |
| Gal3 + 10 uM TD139 vs. Gal3 + 3 uM TD139 | 1.234e-008 | 3.134e-009 | 9.208e-009 | 2.612e-009 | 14 | 8 | 3.525 | 59 |
| Gal3 + 10 uM TD139 vs. Gal3 + 1 uM TD139 | 1.234e-008 | 1.875e-009 | 1.047e-008 | 2.441e-009 | 14 | 10 | 4.289 | 59 |
| Gal3 + 10 uM TD139 vs. 10 uM TD139 | 1.234e-008 | 1.353e-008 | -1.184e-009 | 2.876e-009 | 14 | 6 | 0.4115 | 59 |
| Gal3 + 3 uM TD139 vs. Gal3 + 1 uM TD139 | 3.134e-009 | 1.875e-009 | 1.259e-009 | 2.796e-009 | 8 | 10 | 0.4502 | 59 |
| Gal3 + 3 uM TD139 vs. 10 uM TD139 | 3.134e-009 | 1.353e-008 | -1.039e-008 | 3.183e-009 | 8 | 6 | 3.264 | 59 |
| Gal3 + 1 uM TD139 vs. 10 uM TD139 | 1.875e-009 | 1.353e-008 | -1.165e-008 | 3.044e-009 | 10 | 6 | 3.827 | 59 |

**Supplementary Table 2. Summary of statistics performed in Fig. 1D.**

| Number of families | 1 |  |  |  |  |  |  |  |
| --- | --- | --- | --- | --- | --- | --- | --- | --- |
| Number of comparisons per family | 15 |  |  |  |  |  |  |  |
| Alpha | 0.05 |  |  |  |  |  |  |  |
| **Holm-Sidak's multiple comparisons test** | **Mean Diff.** | **Significant?** | **Summary** | **Adjusted P Value** |  |  |  |  |
| Control KA vs. Gal3 | 0.08364 | Yes | ** | 0.0016 |  |  |  |  |
| Control KA vs. Gal3 + 10 uM TD139 | -0.002866 | No | ns | 0.9153 |  |  |  |  |
| Control KA vs. Gal3 + 3 uM TD139 | 0.02923 | No | ns | 0.7041 |  |  |  |  |
| Control KA vs. Gal3 + 1 uM TD139 | 0.03862 | No | ns | 0.4516 |  |  |  |  |
| Control KA vs. 10 uM TD139 | -0.02401 | No | ns | 0.8267 |  |  |  |  |
| Gal3 vs. Gal3 + 10 uM TD139 | -0.08650 | Yes | ** | 0.0011 |  |  |  |  |
| Gal3 vs. Gal3 + 3 uM TD139 | -0.05440 | No | ns | 0.2627 |  |  |  |  |
| Gal3 vs. Gal3 + 1 uM TD139 | -0.04502 | No | ns | 0.3822 |  |  |  |  |
| Gal3 vs. 10 uM TD139 | -0.1076 | Yes | ** | 0.0016 |  |  |  |  |
| Gal3 + 10 uM TD139 vs. Gal3 + 3 uM TD139 | 0.03210 | No | ns | 0.6847 |  |  |  |  |
| Gal3 + 10 uM TD139 vs. Gal3 + 1 uM TD139 | 0.04149 | No | ns | 0.4402 |  |  |  |  |
| Gal3 + 10 uM TD139 vs. 10 uM TD139 | -0.02114 | No | ns | 0.8267 |  |  |  |  |
| Gal3 + 3 uM TD139 vs. Gal3 + 1 uM TD139 | 0.009385 | No | ns | 0.9153 |  |  |  |  |
| Gal3 + 3 uM TD139 vs. 10 uM TD139 | -0.05324 | No | ns | 0.4402 |  |  |  |  |
| Gal3 + 1 uM TD139 vs. 10 uM TD139 | -0.06263 | No | ns | 0.2627 |  |  |  |  |
| **Test details** | **Mean 1** | **Mean 2** | **Mean Diff.** | **SE of diff.** | **n1** | **n2** | **t** | **DF** |
| Control KA vs. Gal3 | 0.8309 | 0.7473 | 0.08364 | 0.02032 | 14 | 13 | 4.116 | 59 |
| Control KA vs. Gal3 + 10 uM TD139 | 0.8309 | 0.8338 | -0.002866 | 0.01994 | 14 | 14 | 0.1437 | 59 |
| Control KA vs. Gal3 + 3 uM TD139 | 0.8309 | 0.8017 | 0.02923 | 0.02338 | 14 | 8 | 1.250 | 59 |
| Control KA vs. Gal3 + 1 uM TD139 | 0.8309 | 0.7923 | 0.03862 | 0.02184 | 14 | 10 | 1.768 | 59 |
| Control KA vs. 10 uM TD139 | 0.8309 | 0.8549 | -0.02401 | 0.02574 | 14 | 6 | 0.9326 | 59 |
| Gal3 vs. Gal3 + 10 uM TD139 | 0.7473 | 0.8338 | -0.08650 | 0.02032 | 13 | 14 | 4.257 | 59 |
| Gal3 vs. Gal3 + 3 uM TD139 | 0.7473 | 0.8017 | -0.05440 | 0.02371 | 13 | 8 | 2.295 | 59 |
| Gal3 vs. Gal3 + 1 uM TD139 | 0.7473 | 0.7923 | -0.04502 | 0.02219 | 13 | 10 | 2.029 | 59 |
| Gal3 vs. 10 uM TD139 | 0.7473 | 0.8549 | -0.1076 | 0.02604 | 13 | 6 | 4.134 | 59 |
| Gal3 + 10 uM TD139 vs. Gal3 + 3 uM TD139 | 0.8338 | 0.8017 | 0.03210 | 0.02338 | 14 | 8 | 1.373 | 59 |
| Gal3 + 10 uM TD139 vs. Gal3 + 1 uM TD139 | 0.8338 | 0.7923 | 0.04149 | 0.02184 | 14 | 10 | 1.899 | 59 |
| Gal3 + 10 uM TD139 vs. 10 uM TD139 | 0.8338 | 0.8549 | -0.02114 | 0.02574 | 14 | 6 | 0.8213 | 59 |
| Gal3 + 3 uM TD139 vs. Gal3 + 1 uM TD139 | 0.8017 | 0.7923 | 0.009385 | 0.02503 | 8 | 10 | 0.3750 | 59 |
| Gal3 + 3 uM TD139 vs. 10 uM TD139 | 0.8017 | 0.8549 | -0.05324 | 0.02849 | 8 | 6 | 1.869 | 59 |
| Gal3 + 1 uM TD139 vs. 10 uM TD139 | 0.7923 | 0.8549 | -0.06263 | 0.02724 | 10 | 6 | 2.299 | 59 |

**Supplementary Table 3. Summary of statistics performed in Fig. 5E.**

| Number of families | 1 |  | | |  |
| --- | --- | --- | --- | --- | --- |
| Number of comparisons per family | 6 |  |  |  |  |
| Alpha | 0.05 |  |  |  |  |
|  |  |  |  |  |  |
| **Dunn's multiple comparisons test** | **Mean rank diff.** | **Significant?** | **Summary** | **Adjusted P Value** |  |
|  |  |  |  |  |  |
| WT vs. 5xFAD | 21.00 | Yes | ** | 0.0034 |  |
| WT vs. 5xFAD-Gal3KO | 1.995 | No | ns | > 0.9999 |  |
| WT vs. Gal3KO | -1.660 | No | ns | > 0.9999 |  |
| 5xFAD vs. 5xFAD-Gal3KO | -19.00 | Yes | * | 0.0140 |  |
| 5xFAD vs. Gal3KO | -22.66 | Yes | *** | 0.0002 |  |
| 5xFAD-Gal3KO vs. Gal3KO | -3.656 | No | ns | > 0.9999 |  |
| **Test details** | **Mean rank 1** | **Mean rank 2** | **Mean rank diff.** | **n1** | **n2** |
| WT vs. 5xFAD | 29.55 | 8.545 | 21.00 | 11 | 11 |
| WT vs. 5xFAD-Gal3KO | 29.55 | 27.55 | 1.995 | 11 | 10 |
| WT vs. Gal3KO | 29.55 | 31.21 | -1.660 | 11 | 17 |
| 5xFAD vs. 5xFAD-Gal3KO | 8.545 | 27.55 | -19.00 | 11 | 10 |
| 5xFAD vs. Gal3KO | 8.545 | 31.21 | -22.66 | 11 | 17 |
| 5xFAD-Gal3KO vs. Gal3KO | 27.55 | 31.21 | -3.656 | 10 | 17 |

**Supplementary Table 4. Summary of statistics performed in Fig. 5F.**

| Number of families | 1 |  | | | | | | |
| --- | --- | --- | --- | --- | --- | --- | --- | --- |
| Number of comparisons per family | 6 |  |  |  |  |  |  |  |
| Alpha | 0.05 |  |  |  |  |  |  |  |
| **Holm-Sidak's multiple comparisons test** | **Mean Diff.** | **Significant?** | **Summary** | **Adjusted P Value** |  | | | |
| WT vs. 5xFAD | 3.582 | Yes | * | 0.0352 |  |  |  |  |
| WT vs. 5xFAD-Gal3KO | 1.734 | No | ns | 0.4853 |  |  |  |  |
| WT vs. Gal3KO | 1.343 | No | ns | 0.4853 |  |  |  |  |
| 5xFAD vs. 5xFAD-Gal3KO | -1.848 | No | ns | 0.4853 |  |  |  |  |
| 5xFAD vs. Gal3KO | -2.239 | No | ns | 0.2376 |  |  |  |  |
| 5xFAD-Gal3KO vs. Gal3KO | -0.3914 | No | ns | 0.7373 |  |  |  |  |
| **Test details** | **Mean 1** | **Mean 2** | **Mean Diff.** | **SE of diff.** | **n1** | **n2** | **t** | **DF** |
| WT vs. 5xFAD | 28.16 | 24.58 | 3.582 | 1.240 | 11 | 11 | 2.888 | 45 |
| WT vs. 5xFAD-Gal3KO | 28.16 | 26.43 | 1.734 | 1.271 | 11 | 10 | 1.364 | 45 |
| WT vs. Gal3KO | 28.16 | 26.82 | 1.343 | 1.126 | 11 | 17 | 1.193 | 45 |
| 5xFAD vs. 5xFAD-Gal3KO | 24.58 | 26.43 | -1.848 | 1.271 | 11 | 10 | 1.454 | 45 |
| 5xFAD vs. Gal3KO | 24.58 | 26.82 | -2.239 | 1.126 | 11 | 17 | 1.989 | 45 |
| 5xFAD-Gal3KO vs. Gal3KO | 26.43 | 26.82 | -0.3914 | 1.159 | 10 | 17 | 0.3376 | 45 |

**Supplementary Table 5. Summary of statistics performed in Fig. 5E-F for frequency variance shown in supplementary figure 9.**

| Number of families | 1 |  |  |  |  |
| --- | --- | --- | --- | --- | --- |
| Number of comparisons per family | 6 |  |  |  |  |
| Alpha | 0.05 |  |  |  |  |
|  |  |  |  |  |  |
| **Dunn's multiple comparisons test** | **Mean rank diff.** | **Significant?** | **Summary** | **Adjusted P Value** |  |
|  |  |  |  |  |  |
| WT vs. 5xFAD | -20.01 | Yes | ** | 0.0064 |  |
| WT vs. 5xFAD-Gal3KO | -0.009091 | No | ns | > 0.9999 |  |
| WT vs. Gal3KO | -6.321 | No | ns | > 0.9999 |  |
| 5xFAD vs. 5xFAD-Gal3KO | 20.00 | Yes | ** | 0.0084 |  |
| 5xFAD vs. Gal3KO | 13.69 | No | ns | 0.0849 |  |
| 5xFAD-Gal3KO vs. Gal3KO | -6.312 | No | ns | > 0.9999 |  |
|  |  |  |  |  |  |
|  |  |  |  |  |  |
| **Test details** | **Mean rank 1** | **Mean rank 2** | **Mean rank diff.** | **n1** | **n2** |
|  |  |  |  |  |  |
| WT vs. 5xFAD | 18.09 | 38.10 | -20.01 | 11 | 10 |
| WT vs. 5xFAD-Gal3KO | 18.09 | 18.10 | -0.009091 | 11 | 10 |
| WT vs. Gal3KO | 18.09 | 24.41 | -6.321 | 11 | 17 |
| 5xFAD vs. 5xFAD-Gal3KO | 38.10 | 18.10 | 20.00 | 10 | 10 |
| 5xFAD vs. Gal3KO | 38.10 | 24.41 | 13.69 | 10 | 17 |
| 5xFAD-Gal3KO vs. Gal3KO | 18.10 | 24.41 | -6.312 | 10 | 17 |
